# Spatiotemporal evolution and coupling of “Forest–Cropland–Water” ecosystem vulnerability in the Yangtze River Economic Belt

**DOI:** 10.1371/journal.pone.0360810

**Published:** 2026-10-13

**Authors:** Shuai Wu, Xuanhui Li, Xiaohuang Liu, Guanzhong Zeng, Wei Zhang, Xiaojing Liu, Qinghua Zeng, Jie Sun, Liang Ru, Hang Xing, Jiankun Kang

**Affiliations:** 1 Center for Geophysical Survey, China Geological Survey, Langfang, China; 2 Technology Innovation Center for Earth Near Surface Detection, China Geological Survey, Langfang, China; 3 Key Laboratory of Coupling Process and Effect of Natural Resources Elements, Beijing, China; 4 Integrated Natural Resources Survey Center, China Geological Survey, Beijing, China; Universidade Federal de Uberlandia, BRAZIL

## Abstract

The Yangtze River Economic Belt (YREB), China’s “golden waterway” and “green barrier”, relies on its “Forest–Cropland–Water” system for carbon sequestration, grain production, and water conservation. Tracking its vulnerability and coordination on a spatiotemporal scale reveals barriers to balancing ecological security with sustainable growth. Our study innovatively proposes a multi-system coupling evaluation framework. We integrate the Order relation (G1) method, the CRiteria Importance Through Intercriteria Correlation (CRITIC) method, and a game theory combination weighting to assess the ecological vulnerability of the “Forest–Cropland–Water” system across YREB. Using coupling coordination and spatial autocorrelation models, we then map the spatiotemporal pattern and spatial clustering characteristics of ecosystems interactions. Finally, geodetector method is employed to identify the principal drivers of coupling coordination. In 2000–2023, the ecological vulnerability of each ecosystem and the overall system exhibited a fluctuating downward trend. Spatial clustering of coupling coordination gradually weakened, and coupling types shifted from mild imbalance toward mild coordination. Concurrently, high vulnerability areas progressively transitioned to moderate and low vulnerability. Among the driving factors, Land use intensity and aerosol optical depth consistently exhibited the strongest explanatory power for the coupling coordination degree of the “Forest–Cropland–Water” system, whereas the influence of extreme high-temperature days markedly declined over time and the remaining selected factors played only subsidiary roles. This work provides a systematic framework for assessing future multi-system ecological vulnerability and coupling relationship, offering scientific guidance for promoting sustainable natural-resource management and inter-basin development.

## 1 Introduction

Forest, cropland, and water, as essential components of the ecosystem, are indispensable ecological buffers and material foundations for human survival and development, playing an irreplaceable role in both environmental sustainability and economic growth [1–5]. As one of the main components of terrestrial ecosystems, forest systems play a crucial role in key ecological processes such as global climate regulation, environmental protection, and biodiversity conservation [6–8]. The cropland system is fundamental to maintaining agricultural productivity and ensuring food security. Simultaneously, through functions such as soil carbon sequestration, biodiversity preservation, and regional climate regulation, it supports the stability and balance of terrestrial ecosystems [9,10]. The water system is the lifeline of the Earth’s biosphere, providing essential ecosystem including water supply, pollution purification, climate regulation, and aquatic habitat maintenance, while driving the global cycles of matter and energy [11,12]. The forest, cropland, and water systems are closely interconnected through material cycles, energy flows, and ecosystem functions [13]. The forest’s functions of water conservation and climate regulation directly influence cropland productivity and water system stability [14]. As cropland represents areas of intensive human activity, its management practices can exacerbate soil erosion or pollution, threatening the health of both forest and water systems; meanwhile, the spatiotemporal distribution of water resources constrains the ecological carrying capacity of forests and croplands. The dynamic coupling among these three systems determines the overall resilience of regional ecosystems.

Ecological vulnerability refers to the integrated characterization of an ecosystem’s sensitivity to external disturbances and its resilience or recovery capacity within a specific spatiotemporal context [15–17]. Against the backdrop of global climate change and intensified human activities, the assessment of ecological vulnerability has become a key research focus in environmental protection and sustainable development [18]. At present, numerous studies have focused on the ecological vulnerability of forest, cropland, and water systems. For example, Meru and Pandey [19] examined the vulnerability of forest ecosystems in northeastern India under climate change; Li et al. [20] analyzed the spatiotemporal evolution of cropland vulnerability during urbanization in the Huaihai Economic Zone at the county scale; whereas Chen and Feng [21] evaluated and predicted water resource vulnerability in the Yellow River Basin using an RS-SVR model.

However, existing studies are largely confined to single-system scales. The ecological vulnerability of individual systems may be amplified through inter-system interactions, leading to compound ecological risks; for instance, forest degradation can trigger soil erosion, reduce cropland fertility, and exacerbate water scarcity [22]. Therefore, a comprehensive assessment of ecological vulnerability is crucial for identifying the overall quality and vulnerability status of ecosystems.

YREB is located in China’s central golden corridor, spanning the eastern, central, and western regions, and serves as a key engine driving the country’s socio-economic development as well as a strategic barrier for ecological security [23]. Since YREB concept was proposed, the region has experienced rapid economic development. However, factors such as human production activities and environmental pollution have increased the vulnerability of certain ecosystems [24]. In recent years, numerous scholars have conducted extensive research on the ecological environment of YREB and its coupling with economic development. For example, Liu et al. [25] analyzed the coupling and coordinated development of energy, economy, and ecosystems in YREB from 2008 to 2017 based on the DPSIR model; Liu et al. [26] constructed a social–water–environment system coupling model to assess provincial-level coupling coordination in the region; and Ye et al. [27] developed an evaluation system integrating logistics, urbanization, and ecological environment to study the spatiotemporal evolution of coupling coordination among the three systems [27]. However, these studies primarily focus on the macro level and lack quantitative investigations into the internal interactions of natural systems and their driving factors. Therefore, clarifying the coupling mechanisms among different ecosystem vulnerabilities within the natural environment is urgently needed for regional ecological protection.

Forests, croplands, and water, as indispensable material foundations for regional development, play a crucial role in the socio-economic progress of a region. Their degree of balance and coordination is a key determinant of regional ecosystem stability and economic development [28,29]. YREB features extensive forest, cropland areas, and a well-developed water system, with the combined area of these three ecosystems accounting for approximately 80% of the total regional area. Evaluating and analyzing the vulnerability and coupling relationships among these ecosystems is crucial for assessing regional ecological carrying capacity and promoting the balanced development of different ecological systems. Against this background, this study focuses on the YREB and innovatively proposes a multi-system coupling evaluation framework. Using the G1–CRITIC–game theory integrated weighting method, the ecological vulnerability and coupling coordination types of the “Forest–Cropland–Water” system in the YREB were constructed. From a provincial perspective, the coupling relationships among the three ecosystems were analyzed, and their interactive mechanisms were explored. Moreover, spatial autocorrelation models are applied to investigate their spatial clustering characteristics. Geodetector method was employed to identify the primary drivers influencing the spatiotemporal variations in the coupling coordination of the three systems. This study aimed to provide scientific reference for implementing holistic and integrated ecosystem management in the YREB, thereby promoting harmonious development between regional ecological environment and socio-economic systems. Additionally, we proposed a multi-system coupled ecological vulnerability evaluation model. Based on its results, a composite system coupling and coordination framework was constructed. The technical route is illustrated in Fig 1.

**Fig 1:**
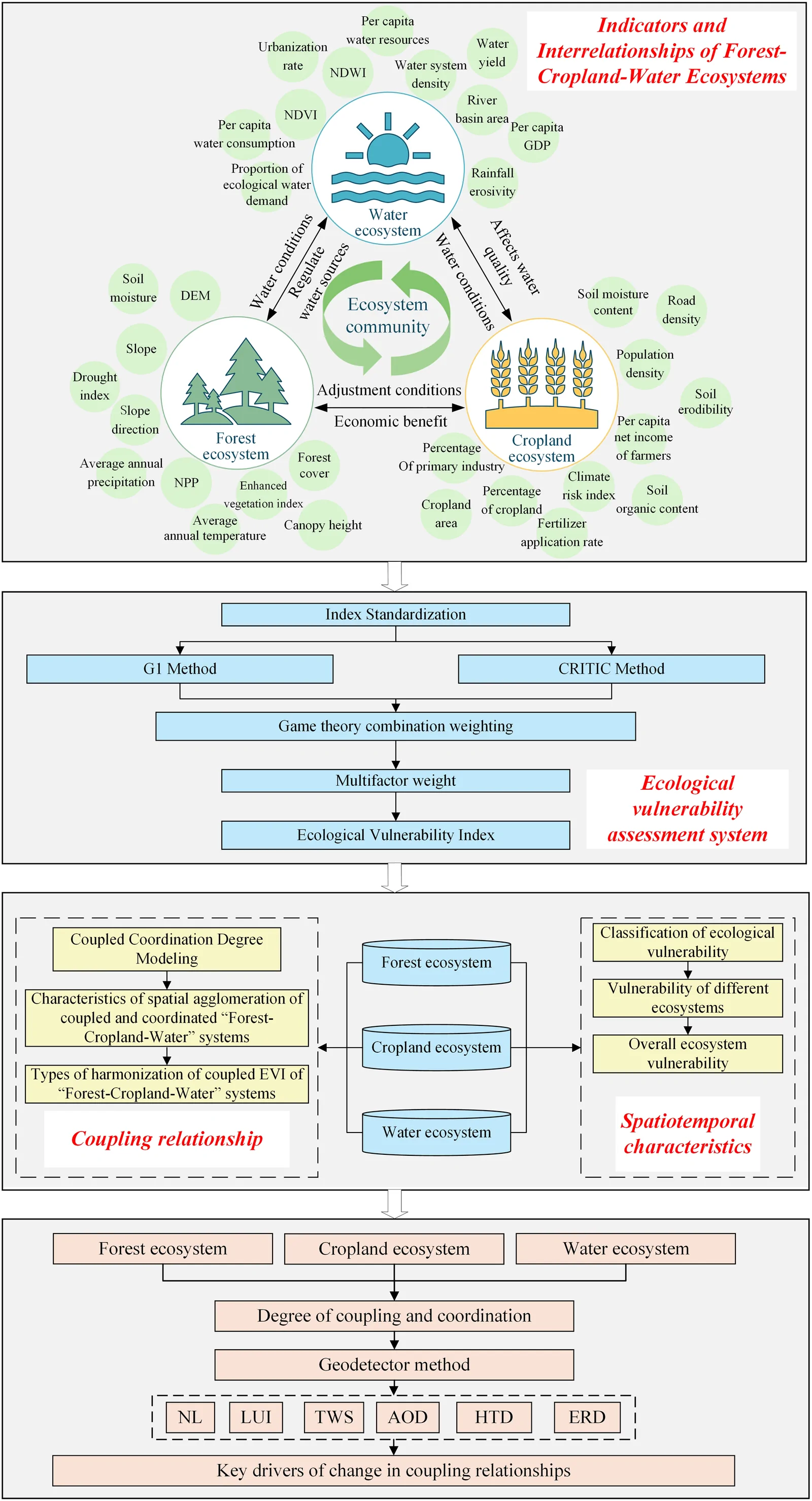
Technical route.

## 2 Materials and methods

### 2.1 Overview of the study area

YREB serves as a critical engine driving China’s socio-economic development. It spans 11 provinces and municipalities, including Yunnan, Sichuan, Chongqing, Guizhou, Hunan, Hubei, Jiangxi, Zhejiang, Anhui, Jiangsu, and Shanghai (Fig 2). Its core is composed of three major economic clusters: the Yangtze River Delta, the middle reaches of the Yangtze River, and the Chengdu–Chongqing urban agglomeration. The region hosts a population of over 600 million, accounting for more than 40% of China’s total population, and contributes more than 40% of the national GDP, supported by a strong industrial base. Oriented along the Yangtze River, the YREB covers a total area of approximately 2.05 × 10^6^ km^2^. Most of the basin lies within the subtropical monsoon climate zone, characterized by four distinct seasons, hot and rainy summers, and mild, relatively dry winters, with an average annual precipitation of about 800–1600 mm. The terrain generally slopes from the highlands in the west to the lowlands in the east, encompassing plateaus, plains, and basins. As a key comprehensive economic zone, the YREB is one of the world’s most integrated industrial corridors, with the most complete industrial system, the strongest influence, and the greatest development potential.

**Fig 2:**
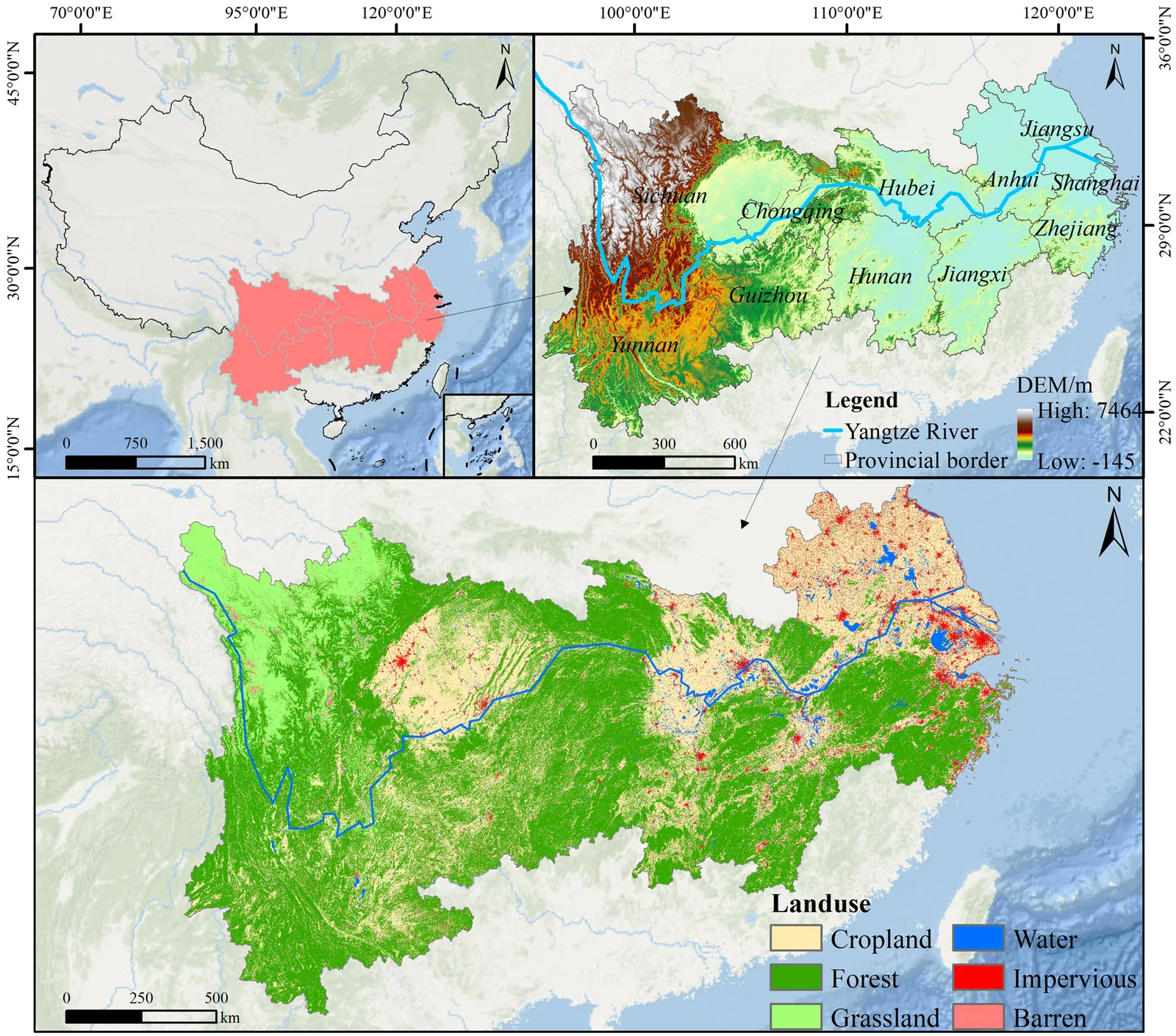
Overview of the study area.

### 2.2 Data sources and processing

This study selected multi-source data reflecting the ecological vulnerability of forest, cropland, and water systems in YREB, with specific indicator details presented in Table 1. Among them, the NDVI data were processed using the maximum value composite method to obtain the annual maximum for the region [30]. Drought index was calculated as the ratio of the difference between potential evaporation and precipitation to potential evapotranspiration [31] and rainfall erosivity was calculated using the established Wischmeier equation [32,33]. Water yield was calculated using the water yield module of the InVEST model based on land use data. Socioeconomic data were obtained from the statistical yearbooks of the study area for 2000–2023. To ensure research scale consistency, socioeconomic data were rasterized using the inverse distance weighting (IDW) interpolation method. Due to differences in spatial resolution and coordinate systems among various datasets, all raster indicators were resampled to a 300 m resolution, with the projection unified as CGCS2000 3° GK Zone 36 to ensure result accuracy. All maps in this document were created using standard maps downloaded from the Ministry of Natural Resources’ Standard Map Service website, with map approval number GS(2024) 0650; the base maps have not been modified.

**Table 1:** Data sources and content.

Data Types	Data Content	Data Sources
NDVI	MOD13Q1 (NASA)	Institute of Tibetan Plateau Research Chinese Academy of Sciences (https://data.tpdc.ac.cn) [34]
NPP	MOD17A3H (NASA)	Google Earth Engine (GEE) (https://lpdaac.usgs.gov/products/mod17a3hgfv061) [35]
NDWI	MOD09GA (NASA)	Google Earth Engine (GEE) ee.ImageCollection(MODIS/061/MOD09GA) (https://code.earthengine.google.com/)
Terrain	ASTER GDEM	Geospatial Data Cloud (http://www.gsc.Loud.cn/)
Enhanced vegetation index	MOD13A2 (NASA)	Resource and Environmental Science Data Platform (https://www.resdc.cn/DOI/doi.aspx?DOIid=49) [36]
Land use	CLCD Land Use	Zenodo(https://zenodo.org/records/12779975) [37]
Crown height	—	Zenodo(https://doi.org/10.5281/zenodo.12697784) [38]
Soil moisture	TerraClimate	TerraClimate (https://www.climatologylab.org)
Forest cover	—	Zenodo (https://zenodo.org/records/11047925) [39]
Soil erodibility	—	(https://doi.org/10.1016/j.scitotenv.2023.168249) [40]
Climate risk index	—	(https://figshare.com/articles/dataset/Climate_Physical_Risk_Index_CPRI_/25562229/3) [41]
Soil organic matter content	—	Institute of Tibetan Plateau Research Chinese Academy of Sciences (https://data.tpdc.ac.cn) [42]
soil moisture content	—	ISRIC (https://data.isric.org/geonetwork/srv/chi/catalog.search#/home) [43]
River basin area	—	Zenodo (https://zenodo.org/records/6895820) [44]
Hydrological	precipitation, temperature	Institute of Tibetan Plateau Research Chinese Academy of Sciences (https://data.tpdc.ac.cn) [45–48]
Road density	—	Science Data Bank (https://cstr.cn/31253.11.sciencedb.16002) [49]
Water system density	—	Science Data Bank (https://cstr.cn/31253.11.sciencedb.j00001.00759) [50]
Soil type	—	Harmonized World Soil Database (https://gaez.fao.org/pages/hwsd)
Population	—	LandScan Global (https://landscan.ornl.gov/)
Socio-economic	Socio-economic indicators from 2000 to 2023	Provincial and municipal statistical yearbooks
Nighttime lighting (NL)	—	Institute of Tibetan Plateau Research Chinese Academy of Sciences (https://data.tpdc.ac.cn) [51]
Land use intensity (LUI)	—	(https://zenodo.org/records/18938572) [52]
Terrestrial water storage (TWS)	—	Institute of Tibetan Plateau Research Chinese Academy of Sciences (https://data.tpdc.ac.cn) [53]
Extreme rainfall (ERD); Extreme high temperature (HTD)	—	(https://figshare.com/articles/dataset/Climate_Physical_Risk_Index_CPRI_/25562229/3) [41]
Aerosol optical depth (AOD)	—	(https://zenodo.org/records/15879684) [54]

### 2.3 Research methods

#### 2.3.1 Evaluation indicator system.

Given that ecosystems themselves constitute complex wholes formed by nonlinear coupling and the interplay of multiple processes, the selection of indicators for assessing ecological vulnerability should prioritize the reflection of interactions among elements. In this study, the indicators for evaluating the “Forest–Cropland–Water” system were selected based on the actual conditions of the study area, with reference to relevant indicator systems in ecological vulnerability research. Following the principles of scientific rigor and systematicity, eleven of the most representative indicators were chosen for each of the three subsystems, ensuring that the selected indicators adequately reflect the state and key ecological processes of the ecosystem while also capturing the interactive relationships among the indicators (Table 2). Indicator characteristics: Positive indicate that higher values correspond to higher ecological vulnerability, whereas negative indicate the opposite.

**Table 2:** Coupled ecosystem vulnerability indicator system.

Principles layer	Indicator layer	Resolution	Temporal resolutions	Indicator characteristics	Ecological significance
Forest system	Slpoe (A1)	30m	–	Positive	Terrain Stability and Erosion Risk
	DEM (A2)	30m	–	Positive	Terrain Stability and Erosion Risk
	Slope direction (A3)	30m	–	Positive	Vegetation Light–Moisture Variability
	Drought index (A4)	1000m	Year	Positive	Water-Balance Stress on Vegetation Growth
	Net Primary Production (NPP) (A5)	500m	Year	Negative	Ecosystem Productivity Indicator
	Enhanced vegetation index (A6)	1000m	Year	Negative	Vegetation Cover Dynamics
	Annual average temperature (A7)	1000m	Month	Positive	Hydrothermal Conditions for Vegetation Growth
	Annual precipitation (A8)	1000m	Month	Negative	Hydrothermal Conditions for Vegetation Growth
	Canopy height (A9)	30m	–	Negative	Ecosystem Structural Stability
	Soil moisture (A10)	4000m	Year	Negative	Key Limiting Factors for Vegetation Growth
	Forest cover (A11)	30m	Year	Negative	Strength of Ecological Barrier Function
Cropland system	Population density (B1)	1000m	Year	Positive	Intensity of Human Disturbance
	Cropland area (B2)	City scale	Year	Negative	Degree of Land-Use Intensification
	Percentage of cropland (B3)	City scale	Year	Negative	Degree of Land-Use Intensification
	Road density (B4)	1000m	–	Negative	Frequency of Economic Activities
	Percentage of primary industry (B5)	City scale	Year	Negative	Agricultural Economic Development
	per capita net income of farmers (B6)	Provincial scale	Year	Negative	Economic Basis for cropland Protection
	Soil erodibility (B7)	1000m	–	Positive	Soil Erosion Status
	Climate risk index (B8)	City scale	Year	Positive	Frequency of Extreme Weather Events
	Soil organic matter content (B9)	1000m	–	Positive	Soil Fertility and Carbon-Sink Capacity
	Fertilizer application (B10)	Provincial scale	Year	Positive	Potential Nonpoint-Source Pollution Risk
	Soil moisture content (B11)	250m	Year	Negative	Drought Resilience of Cropland
Water system	Per capita water consumption (C1)	Provincial scale	Year	Negative	Water Consumption Pressure Index
	Per capita water resources (C2)	Provincial scale	Year	Negative	Water Supply–Demand Conflict Index
	Rainfall erosivity (C3)	1000m	Year	Positive	Potential for Soil and Water Loss
	Normalized Difference Vegetation Index (NDVI) (C4)	250m	Month	Negative	Vegetation Water-Conservation Response
	Water system density (C5)	1000m	–	Negative	Hydrological Regulation Capacity
	Normalized Difference Water Index (NDWI) (C6)	500m	Year	Negative	Surface Water Body Distribution
	River basin area (C7)	1000m	–	Negative	Hydrological Connectivity
	Proportion of ecological water demand (C8)	Provincial scale	Year	Positive	Water Resource Competition Intensity
	Per capita GDP (C9)	City scale	Year	Positive	Water Use Intensity for Economic Development
	Urbanization rate (C10)	Provincial scale	Year	Positive	Pressure from Anthropogenic Expansion
	Water yield (C11)	30m	Year	Negative	Water Source Conservation Capacity

In forest ecosystems, slope reflects the surface inclination degree; steep slopes are prone to soil erosion, which in turn weakens vegetation recovery capacity [55]. Elevation influences vegetation types through variations in temperature, precipitation, and solar radiation; in high-altitude areas, harsh climatic conditions and prolonged recovery cycles further exacerbate ecological fragility [56,57]. Slope direction determines microclimatic conditions, with sunny slopes generally exhibiting more favorable heat and moisture availability, while shady slopes are characterized by limited solar radiation and lower temperatures [56]. Among climatic indicators, the drought index quantifies the balance between water supply and demand, and at the macroscale, drought levels determines the zonal distribution pattern of vegetation: with higher drought levels, vegetation types transition from tall forests in humid areas to sparse shrubs or grasslands in arid areas, while biomass gradually decreases, thereby weakening ecological carrying capacity [56]. Annual anomalies in temperature and precipitation disrupt ecological balance, as elevated temperatures accelerate evapotranspiration, while insufficient rainfall leads to soil drought [55]. In terms of vegetation function, Net Primary Production (NPP) reflects the productivity and cycling capacity of biological communities, thereby influencing their energy flow and material circulation, and serves as a key parameter for measuring the stability of ecosystems. Lower NPP values indicate weaker system resilience within the biological community and a diminished capacity to resist external disturbances, thus resulting in greater ecological vulnerability [55]. Enhanced Vegetation Index is used to monitor vegetation cover and biomass, where a decline in its value is likely to result in ecological degradation [58]. Canopy height is closely associated with forest maturity; larger canopies indicate stronger carbon sequestration capacity and higher structural stability of the ecosystem [56]. Low forest coverage exacerbates habitat fragmentation and restricts species migration [56]. Soil moisture determines water and nutrient availability; low moisture levels inhibit plant growth and weaken carbon sequestration capacity [59]. In the cropland system, high population density intensifies competition for arable land, leading to overcultivation and ecological overload, which in turn weakens system stability [20]. The larger the cropland area and the higher the degree of scale, the more intensive management reduces soil erosion and resource waste, thereby enhancing the stability of the cropland system, strengthening its capacity to resist external disturbances, and lowering its ecological vulnerability [20]. A higher proportion of cropland reduces land-use fragmentation, preventing scattered development in ecologically vulnerable areas and lowering the risk of external disturbances [20]. Higher road density can fragment habitats, increase pollution, and reduce cropland restoration potential [60]. A higher proportion of the primary industry promotes agricultural specialization and intensification, reducing extensive land exploitation, and mitigating ecological disturbances [25]. Low per capita net income of farmers can lead to extensive farming practices, whereas higher income may promote the adoption of sustainable technologies [61]. Higher soil erodibility makes the soil more susceptible to wind and water erosion, leading to nutrient loss and threatening cropland productivity and sustainability [40]. Climate risk index is a weighted composite of four indicators: days of extreme low temperature, extreme high temperature, extreme rainfall, and extreme drought, and the higher it is, the more frequent extreme weather events directly damage crop growth and destabilize the cropland system [41]. Lower soil organic matter content reduces soil fertility and water retention capacity [42]. Excessive fertilizer application can lead to soil acidification and water pollution, disrupting microbial balance [20]. Insufficient soil moisture content restricts crop growth [62]. In the water system, per capita water consumption reflects the intensity of water resource consumption; high values indicate greater demand pressure, which can easily lead to water cycle imbalances [11]. Per capita water resources measure the endowment of water resources in a region; low values indicate water scarcity risks, intensifying supply-demand conflicts and weakening the system’s capacity to cope with drought and other stresses [11]. Rainfall erosivity is the soil-detaching power of rainfall; in areas with high values, the risk of sediment entering water bodies is elevated, exacerbating river siltation and water pollution [63]. NDVI represents vegetation cover; areas with low values exhibit exposed soil, which can exacerbate soil erosion and water loss [8]. Water system density reflects the river network density; areas with low density have limited water resource allocation capacity [64]. NDWI indicates the surface water distribution and dynamics; a decrease in its value signifies wetland shrinkage or river fragmentation, directly threatening the aquatic ecosystem stability [65]. A larger river basin area implies a wider catchment range and stronger water resource carrying capacity [66]. A high proportion of ecological water demand indicates that human activities are encroaching on the natural water cycle, leading to river discontinuity and aquatic habitat degradation [67]. A higher per capita GDP may elevate industrial water consumption and greater pollutant discharge [68]. High urbanization rates disrupt the hydrological cycle through impervious surfaces, expand the scope of pollution, and encroach upon ecological space, leading to the degradation of water ecosystem functions and thereby increasing their vulnerability [69]. Areas with low water yield are prone to persistent water scarcity due to climate change or overexploitation [67].

#### 2.3.2 G1method.

The G1 method is a subjective weighting approach based on expert judgment, whose core concept lies in replacing the complex judgment matrix of the Analytic Hierarchy Process (AHP) based on importance ratios between adjacent indicators [70]. While retaining the subjective judgment characteristics, this method eliminates the need for the consistency check required in AHP. By adopting a hierarchical comparison mechanism, the rank-order method simplifies the weight calculation process and significantly improves computational efficiency, making it particularly suitable for weight determination and analysis in multi-level decision-making models [57]. The calculation process is described as follows:

(1) The sequence relationship of indicators is determined, specifying their relative importance to the evaluation criteria as *x*_1_ > *x*_2_ > ... > *x*_*m。*_ *X*_1*-m*_ represents the order of indicators.
(2) Based on expert judgment, a rational assessment of the relative importance ratio *r*_*k*_ between adjacent indicators is determined:

$$\begin{array}{c} r_{k}=Y_{k-1}/Y_{k} \end{array}$$ (1)

*Y* represents the relative importance ratio of indicator *x,* and *k* represents the k-th indicator.
(3) The weight coefficients *w*_*k*_ are calculated:

$$\begin{array}{c} W_{k}={\left(1+\sum_{k=2}^{m}\prod_{i=k}^{m}r_{k}\right)}^{-1} \end{array}$$ (2)

$$\begin{array}{c} w_{k-1}=r_{k}w_{k} \end{array}$$ (3)

#### 2.3.3 CRITIC method.

The CRITIC method was originally proposed by Diakoulaki as an objective weighting method [71]. It accounts for the intrinsic information contained within each indicator, effectively capturing the conflict and variability among different alternatives. Compared to the entropy weighting method, CRITIC emphasizes more on the horizontal comparison between indicators, resulting in more accurate and reasonable weight calculations [72]. The calculation is as follows:

(1) First, data is normalized using the specific formula provided in the literature [73].
(2) The standard deviation of each indicator and the correlation coefficients between indicators in the standardized matrix X are expressed below:

$$\begin{array}{c} \omega_{i}=\sqrt{\frac{\text{1}}{m}{\sum_{i=1}^{m}(x_{ij}-\mathrm{\backslash stackrel}\_x_{j})}^{\text{2}}}\begin{array}{cc} & i=1,2,\text{\textbackslash{}ldots},n \end{array} \end{array}$$ (4)

$$\begin{array}{c} \rho_{ij}=cov\left(X_{i},X_{j}\right)/\left(\omega_{i},\omega_{j}\right)\begin{array}{cc} & i,j=1,2,\text{\textbackslash{}ldots},n \end{array} \end{array}$$ (5)

In the formula, *ω*_*j*_ represents the standard deviation of the *j*_*th*_ indicator; *p*_*ij*_ represents the correlation coefficient between the *i*_*th*_ and *j*_*th*_ indicators; and *X*_*_i*_ and *X_*_*j*_ represent the i-th and j-th columns, respectively, of the standardized matrix X.
(3) Indicator information content *W*_*j*_ is defined as follows:

$$\begin{array}{c} W_{j}=\omega_{j}\sum_{i=1}^{n}1-\rho_{ij} \end{array}$$ (6)

(4) The objective weights *E*_*j*_ of the indicators are calculated below:

$$\begin{array}{c} E_{j}=\frac{W_{j}}{\sum_{j=1}^{n}W_{j}} \end{array}$$ (7)

#### 2.3.4 Game theory combination weighting.

This study constructed an integrated combination-weight model based on the principle of game-theoretic equilibrium. By establishing an optimization model that minimizes deviation among individual weighting vectors, the Nash equilibrium solution between the G1 method and the CRITIC method is obtained, resulting in an optimal combined weighting scheme that incorporates both subjective and objective information [74]. This model effectively integrated the expert knowledge advantage of the G1 method with the data-driven characteristics of the CRITIC method. By establishing a game-theoretic coordination mechanism among the weights, it mitigates the potential bias of single weighting approaches and enhances scientific rigor and robustness of the evaluation system, providing a weighting calculation scheme that offers both theoretical soundness with strong practical interpretability for complex decision-making problems [75].

Within the game theory-based comprehensive weighting framework, the set of ecological vulnerability indicator weight vectors ($W=\sum_{k=1}^{N}\alpha_{k}w_{k}^{T}$) generated by N different methods are integrated. By optimizing the combination coefficients *α*_*k*_ to minimize deviation among the weight vectors, a combined weight optimization model is constructed as follows:


$$\begin{array}{c} min\left\|\sum_{k=1}^{N}\alpha_{j}w_{j}^{T}-w_{i}^{T}\right\|(i=1,2,\text{\textbackslash{}ldots},N) \end{array}$$ (8)

Using matrix differentiation properties, Equation (8) is reformulated into a linear equation system representing the first-order optimality conditions:


$$\begin{array}{c} \left[\begin{array}{cccc} @cccc@w_{\text{1}}w_{\text{1}}^{T} & w_{\text{1}}w_{\text{2}}^{T} & \text{\textbackslash{}ldots} & w_{\text{1}}w_{\delta }^{T}\\ w_{\text{2}}w_{\text{1}}^{T} & w_{\text{2}}w_{\text{2}}^{T} & \text{\textbackslash{}ldots} & w_{\text{1}}w_{\delta }^{T}\\ \vdots & \vdots & \vdots & \vdots \\ w_{\delta }w_{\text{1}}^{T} & w_{\delta }w_{\text{2}}^{T} & \text{\textbackslash{}ldots} & w_{\text{1}}w_{\delta }^{T} \end{array}\right]\left[\begin{array}{c} @c@\alpha_{\text{1}}\\ \alpha_{\text{2}}\\ \vdots \\ \alpha_{\delta } \end{array}\right]=\left[\begin{array}{c} @c@w_{\text{1}}w_{\text{1}}^{T}\\ w_{\text{2}}w_{\text{2}}^{T}\\ \vdots \\ w_{\delta }w_{\delta }^{T} \end{array}\right] \end{array}$$ (9)

The final combined weights are obtained by standardizing the weights derived from each weighting method:


$$\begin{array}{c} W=\sum_{k=1}^{M}\alpha_{k}^{*}w_{k}^{T} \end{array}$$ (10)

Based on the Game theory combination weighting, the G1 and CRITIC methods were linearly combined through coefficient optimization, and the resulting weights are presented in Table 3.

**Table 3:** Indicator weights(%).

Criterion layer	Indicator layer	G1 method	CRITIC method	Game theory combination weighting
Forest system	Slpoe	6.575	5.633	6.199
	DEM	25.449	7.749	18.381
	Slope direction	9.468	1.426	6.257
	Drought index	15.906	7.244	12.447
	NPP	13.255	9.482	11.748
	Enhanced vegetation index	3.805	7.786	5.394
	Annual average temperature	3.805	18.535	9.686
	Annual precipitation	7.890	15.847	11.067
	Canopy height	5.479	7.826	6.416
	Soil moisture	4.566	8.381	6.089
	Forest cover	3.805	10.09	6.314
Cropland system	Population density	4.225	6.099	5.177
	Cropland area	4.225	12.667	8.514
	Percentage of cropland	3.520	6.554	5.062
	Road density	7.300	17.87	12.670
	Percentage of primary industry	12.264	12.478	12.373
	per capita net income of farmers	6.083	16.078	11.161
	Soil erodibility	17.660	5.946	11.708
	Climate risk index	5.069	0.798	2.899
	Soil organic matter content	10.220	4.487	7.307
	Fertilizer application	14.717	9.136	11.881
	Soil moisture content	14.717	7.887	11.247
Water system	Per capita water consumption	11.415	8.875	10.966
	Per capita water resources	13.698	4.858	12.137
	Rainfall erosivity	21.917	12.9	20.324
	NDVI	3.932	9.011	4.829
	Water system density	4.719	6.987	5.119
	NDWI	9.513	11.239	9.818
	River basin area	3.932	8.382	4.718
	Proportion of ecological water demand	6.795	12.404	7.786
	Per capita GDP	5.662	12.593	6.887
	Urbanization rate	13.698	7.449	12.594
	Water yield	4.719	5.302	4.822

#### 2.3.5 Ecological vulnerability index.

This study used the Ecological Vulnerability Index (EVI) to analyze and evaluate the forest, cropland, water systems, and overall ecological environment of YREB [76]. Drawing on previous EVI models [77], the calculation formula is as follows:


$$\begin{array}{c} EVI=\sum_{i=1}^{n}a_{j}\times r_{ij} \end{array}$$ (11)

where *a*_*j*_ represents the weight of the *j*-th indicator; *r*_*ij*_ is the normalized indicator value; and *n* denotes the total number of indicators. Based on field conditions and existing classification standards [78,79], the vulnerability results were divided into five levels using an equal-interval method: slight vulnerability [0 ~ 0.2], mild vulnerability (0.2 ~ 0.4], moderate vulnerability (0.4 ~ 0.6], severe vulnerability (0.6 ~ 0.8], and extreme vulnerability (0.8 ~ 1.0].

#### 2.3.6 Coupling coordination degree model.

The coupling coordination degree model is commonly used to assess the interactions and coordination levels among different ecosystems. In this study, a comprehensive coupling coordination vulnerability evaluation model was developed to quantitatively analyze the coupling coordination degree of the “Forest–Cropland–Water” system [80]. The formulas are as follows:


$$\begin{array}{c} C={\left[\frac{F_{i}\times P_{i}\times W_{i}}{{\left[\left(F_{i}+P_{i}+W_{i}\right)/3\right]}^{3}}\right]}^{\frac{1}{3}} \end{array}$$ (12)

$$\begin{array}{c} T=\alpha F_{i}+\omega P_{i}+\beta W_{i} \end{array}$$ (13)

$$\begin{array}{c} D=\sqrt{C\times T} \end{array}$$ (14)

In Equation 12, *C* represents the coupling degree of the “Forest–Cropland–Water” ecological vulnerability system, with *C* = 1 indicating the highest coupling degree, and *α, ω,* and *β* are undetermined coefficients. *F*_*i*_*, P*_*i*_ and *W*_*i*_ represent the ecological vulnerability index for the forest, cropland, and water ecosystems, respectively, in the i-th county or district. This study considered the forest, cropland, and water ecosystems equally important, thus setting *α* = *ω* = *β* = 1/3; *n* denotes the number of ecosystems; and *D* is the coupling coordination degree, where a larger value indicates a higher coupling coordination level. *T* represents coordination. Based on existing literature and study area status, the coupling coordination degree was divided into six categories. Combined with the ecological vulnerability levels, the coupling coordination degree was further subdivided into 18 subcategories (Table 4).

**Table 4:** Classification criteria for coupling coordination.

Coupling Coordination Types	Coordination Development Range	Combined Criteria for Ecological Vulnerability Classification	Coupling Coordination Degree Types
Severe imbalance	0 ≤ D < 0.4	0 ≤ EVI < 0.34	Severe imbalance-low vulnerability
		0.34 ≤ EVI < 0.67	Severe imbalance-moderate vulnerability
		0.67 ≤ EVI ≤ 1	Severe imbalance-high vulnerability
Moderate imbalance	0.4 ≤ D < 0.5	0 ≤ EVI < 0.34	Moderate imbalance-low vulnerability
		0.34 ≤ EVI < 0.67	Moderate imbalance-moderate vulnerability
		0.67 ≤ EVI ≤ 1	Moderate imbalance-high vulnerability
Mild imbalance	0.5 ≤ D < 0.6	0 ≤ EVI < 0.34	Mild imbalance-low vulnerability
		0.34 ≤ EVI < 0.67	Mild imbalance-moderate vulnerability
		0.67 ≤ EVI ≤ 1	Mild imbalance-high vulnerability
Mild coordination	0.6 ≤ D < 0.7	0 ≤ EVI < 0.34	Mild coordination-low vulnerability
		0.34 ≤ EVI < 0.67	Mild coordination-moderate vulnerability
		0.67 ≤ EVI ≤ 1	Mild coordination-high vulnerability
Moderate coordination	0.7 ≤ D < 0.8	0 ≤ EVI < 0.34	Moderate coordination-low vulnerability
		0.34 ≤ EVI < 0.67	Moderate coordination-moderate vulnerability
		0.67 ≤ EVI ≤ 1	Moderate coordination-high vulnerability
High coordination	0.8 ≤ D ≤ 1	0 ≤ EVI < 0.34	High coordination-low vulnerability
		0.34 ≤ EVI < 0.67	High coordination-moderate vulnerability
		0.67 ≤ EVI ≤ 1	High coordination-high vulnerability

#### 2.3.7 Spatial autocorrelation.

Spatial autocorrelation refers to the correlation between the attribute value of a given location and those of its neighboring areas in geographic space. It is used to identify local spatial heterogeneity by statistically quantifying the relationships between spatial units and their surroundings, thereby revealing spatial clustering patterns. Spatial autocorrelation can be categorized into global and local types [13]. The study employed Moran’s I and Local Spatial Autocorrelation methods to investigate the spatial clustering characteristics of the coupling coordination degree of the “Forest–Cropland–Water” system in YREB. The formula for Local Spatial Autocorrelation is as follows:


$$\begin{array}{c} I=\frac{n\sum_{i=1}^{n}\sum_{j=1}^{n}w_{ij}\left(y_{i}-\overset{―}{y}\right)\left(y_{j}-\overset{―}{y}\right)}{\sum_{i=1}^{n}\sum_{j=1}^{n}w_{ij}\sum_{i=1}^{n}{\left(y_{i}-y\right)}^{2}} \end{array}$$ (15)

In the formula, *n* is the number of counties and districts, *y*_i_ and *y*_j_ are the attribute values of the *i*-th and *j*-th counties and districts, *y* is the average attribute value, and *w*_ij_ is the spatial weight between the *i*-th and *j*-th counties and districts.

The formula for Moran’s I is as follows:


$$\begin{array}{c} I=\frac{\sum_{i=1}^{n}\sum_{j=1}^{n}w_{ij}k_{i}k_{j}}{\sum_{i=1}^{n}k_{i}^{2}} \end{array}$$ (16)

In the formula, *k*_i_ is the deviation of counties and districts *i* from its average value (*y*_*i–$\overset{―}{y}$*_), *w*_ij_ is the spatial weight of counties and districts *i* and *j*, and *n* is the total number of counties and districts.

#### 2.3.8 Geodetector method.

The geodetector method is a spatial statistical technique employed to detect the spatial heterogeneity of the dependent variable and to quantify the explanatory power of its driving factors, thus proving well suited for exploring complex nonlinear relationships among variables [79]. The coupling coordination of vulnerability in the “Forest–Cropland–Water” system of the YREB is a complex system influenced by multiple factors. Therefore, based on the actual conditions of regional development and the environment, this study selected six indicators—NL, LUI, HTD, ERD, AOD, and TWS—as independent variables to investigate their impacts on the coupling coordination degree of the system from both natural environmental and socio-economic perspectives.


$$\begin{array}{c} q=1-\frac{\sum_{h=1}^{L}N_{h}\sigma_{h}^{\text{2}}}{N\sigma^{\text{2}}} \end{array}$$ (17)

The formula is expressed as follows: where *q* denotes the explanatory power of each indicator with respect to the ecological vulnerability index, with values ranging from 0 to 1; *h* = 1, …, *L* represents the stratification of the dependent or independent variable; *N*_*h*_ and *N* are the numbers of units in stratum *h* and the entire study area, respectively; and *σ*_*h*_^*2*^ and *σ*^*2*^ are the variances within stratum *h* and the whole study area, respectively.

## 3 Results

### 3.1 Spatiotemporal evolution of ecological vulnerability in forest systems

From 2000 to 2023, the ecological vulnerability of the forest system in YREB exhibited an overall fluctuating downward trend (Fig 3). In 2000–2010, vulnerability declined significantly, and the ecological environment continuously improved, with areas of slight and moderate vulnerability expanding steadily; their proportions increased by 14.26 and 0.22%, respectively. In contrast, areas of severe and extreme vulnerability shrank, with proportions decreasing by 7.92% and 7.42%, respectively. From 2010 to 2015, vulnerability increased slightly: the proportions of slight and mild vulnerability declined by 0.86% and 2.06%, respectively, while those of moderate and extreme vulnerability rose by 2.67% and 3.54%, respectively. Between 2015 and 2020, vulnerability levels gradually decreased, with the combined proportion of severe and extreme vulnerability dropping by 7.2%, and the combined proportion of slight and moderate vulnerability increasing by 7.2%. From 2020 to 2023, vulnerability intensified again, as the proportion of slight vulnerability decreased by 5.32%, while those of moderate, severe, and extreme vulnerability increased by 2.36%, 1.91%, and 1.05%, respectively.

**Fig 3:**
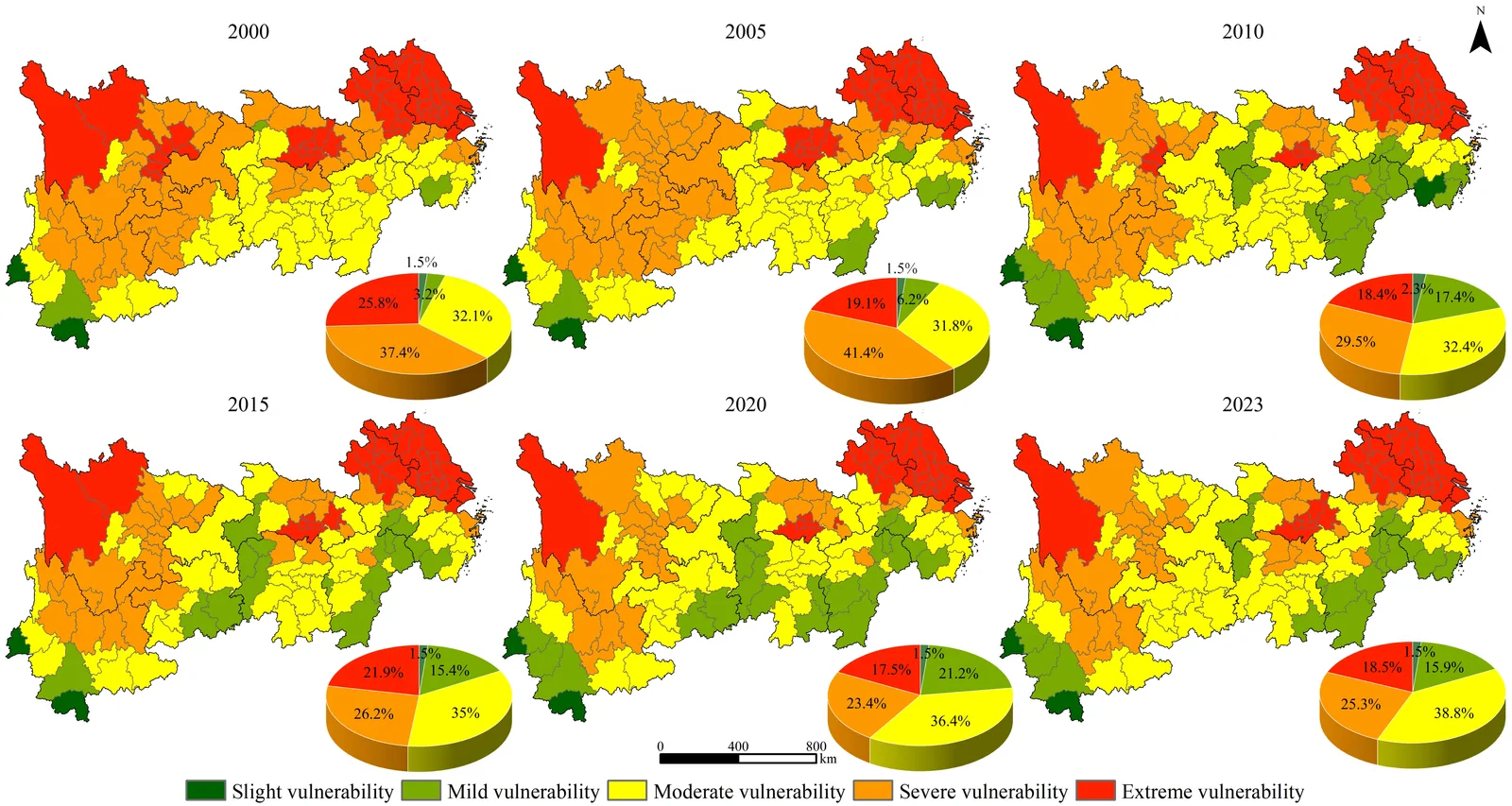
Spatiotemporal distribution of forest system ecological vulnerability.

Spatially, the forest system in YREB exhibited a general pattern of decreasing ecological vulnerability from north to south. Areas of extreme and severe vulnerability showed clustered distributions, primarily concentrated in the western junction region of Sichuan, Yunnan, Guizhou, and Chongqing, the central provinces of Hunan and Hubei, and much of the eastern areas, including Anhui, Jiangsu, and Shanghai. Sichuan and Anhui are situated mainly in transitional zones between mountains and basins, characterized by fragmented terrain and steep slopes, which are prone to soil erosion and geological hazards. The economically developed regions of Hunan, Jiangsu, and Shanghai have high urbanization rates, where the expansion of construction land has caused forest fragmentation. Moreover, due to economic development pressures and strategic priorities, ecological protection efforts in these areas have been relatively insufficient. Moderate vulnerability zones were found across all provinces except Jiangsu and Shanghai. Areas of slight and minimal vulnerability were mainly concentrated in Yunnan, southern Jiangxi, southern Zhejiang, and the junction region of Chongqing, Guizhou, Hubei, and Hunan provinces. Yunnan and southern Zhejiang predominantly lie in gently sloping hills or river valleys with strong soil and water conservation capacity. Jiangxi and Zhejiang benefit from abundant precipitation, high forest coverage, and stable ecosystems. Additionally, higher investment in ecological protection and the effective operation of nature reserves and ecological restoration projects have significantly mitigated the ecological vulnerability of the forest system in these areas.

### 3.2 Spatiotemporal evolution of cropland system ecological vulnerability

From 2000 to 2023, the ecological vulnerability of the cropland system in YREB exhibited alternating declines (Fig 4). Between 2000 and 2005, vulnerability significantly decreased, with the areas of slight, mild, and moderate vulnerability notably expanding; their proportions increased by 1.38%, 10.4%, and 1.67%, respectively. Conversely, areas of severe and extreme vulnerability contracted, with proportions declining by 9.09% and 3.67%, respectively. From 2005 to 2010, vulnerability slightly increased, as the proportions of slight and mild vulnerability decreased by 0.8% and 1.79%, respectively, while those of moderate and severe vulnerability rose by 1.46% and 1.53%, respectively. Between 2010 and 2015, vulnerability marginally declined, mainly reflected in the transition of many severely vulnerable areas to moderate vulnerability. From 2015 to 2020, vulnerability intensified; the moderate vulnerability area shrank, with its proportion decreasing by 17.33%, whereas the severe vulnerability area expanded, increasing by 20.06%. Between 2020 and 2023, overall vulnerability decreased again, with the area of mild vulnerability expanding and its proportion increasing by 6.49%, while the areas of severe and extreme vulnerability sharply contracted, collectively decreasing by 8.44%.

**Fig 4:**
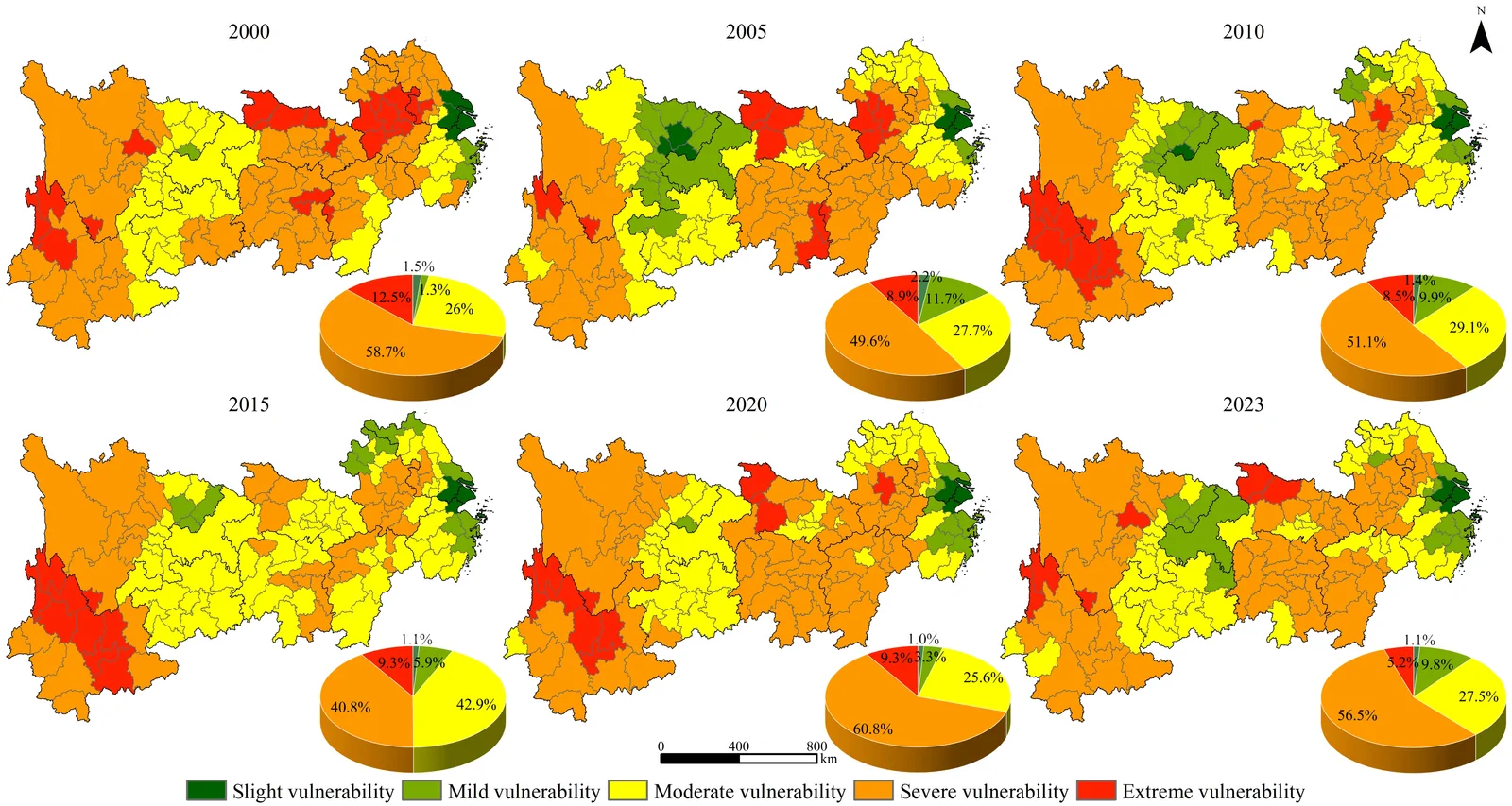
Spatiotemporal distribution of cropland system ecological vulnerability.

Spatially, the cropland system in YREB exhibited a “stepped decrease from west to east” distribution pattern. Areas of extreme and severe vulnerability showed significant spatial clustering, primarily located along the western edge of the Sichuan Basin, the transitional zone of the Yunnan-Guizhou Plateau, and the midstream urban agglomerations in Hubei, Hunan, Jiangxi, and Anhui provinces. The junction between Sichuan and Yunnan is characterized by karst and hilly terrain with shallow soil layers and a high risk of rocky desertification, compounded by frequent seasonal droughts, leading to continuous degradation of cropland quality. Central economic regions such as Hubei and Hunan face rapid industrialization, resulting in pronounced non-agricultural use of cropland, intensified soil heavy metal pollution, and increased ecological vulnerability due to crop rotation obstacles. Moderate vulnerability zones form belts mainly distributed across the central-western and eastern parts of YREB, including Guizhou, northern Jiangsu, and southern Zhejiang. These areas generally have a relatively low soil organic matter content and experience a higher number of extreme weather days, resulting in moderate cropland quality. Slight and minimal vulnerability areas are concentrated around Shanghai and Chongqing. In the Three Gorges Reservoir area of Chongqing, comprehensive management of sloping croplands has significantly reduced soil erosion risks and enhanced soil water retention and erosion resistance. Shanghai has stabilized cropland quality by strictly controlling disorderly urban land expansion, delineating permanent basic cropland protection zones, and integrating cropland protection into urban ecological network planning.

### 3.3 Spatiotemporal evolution of ecological vulnerability in water systems

From 2000 to 2023, the ecological vulnerability of the water system in YREB exhibited a fluctuating downward trend, similar to that of the forest system (Fig 5). From 2000 to 2005, vulnerability declined markedly, characterized by substantial reductions in the proportions of areas with slight, moderate, and extreme vulnerability, which decreased by 17.28%, 2.97%, and 0.22%, respectively. Meanwhile, the proportion of regions with very slight vulnerability increased significantly by 20.25%. From 2005 to 2015, vulnerability gradually intensified, with the proportion of very slightly vulnerable areas decreasing by 16.73%, while that of severely vulnerable areas increased by 3.52%. From 2015 to 2023, vulnerability continued to decline, with the proportion of very slightly vulnerable areas rising by 12.21% and that of slightly vulnerable areas decreasing by 16.38% over eight years.

**Fig 5:**
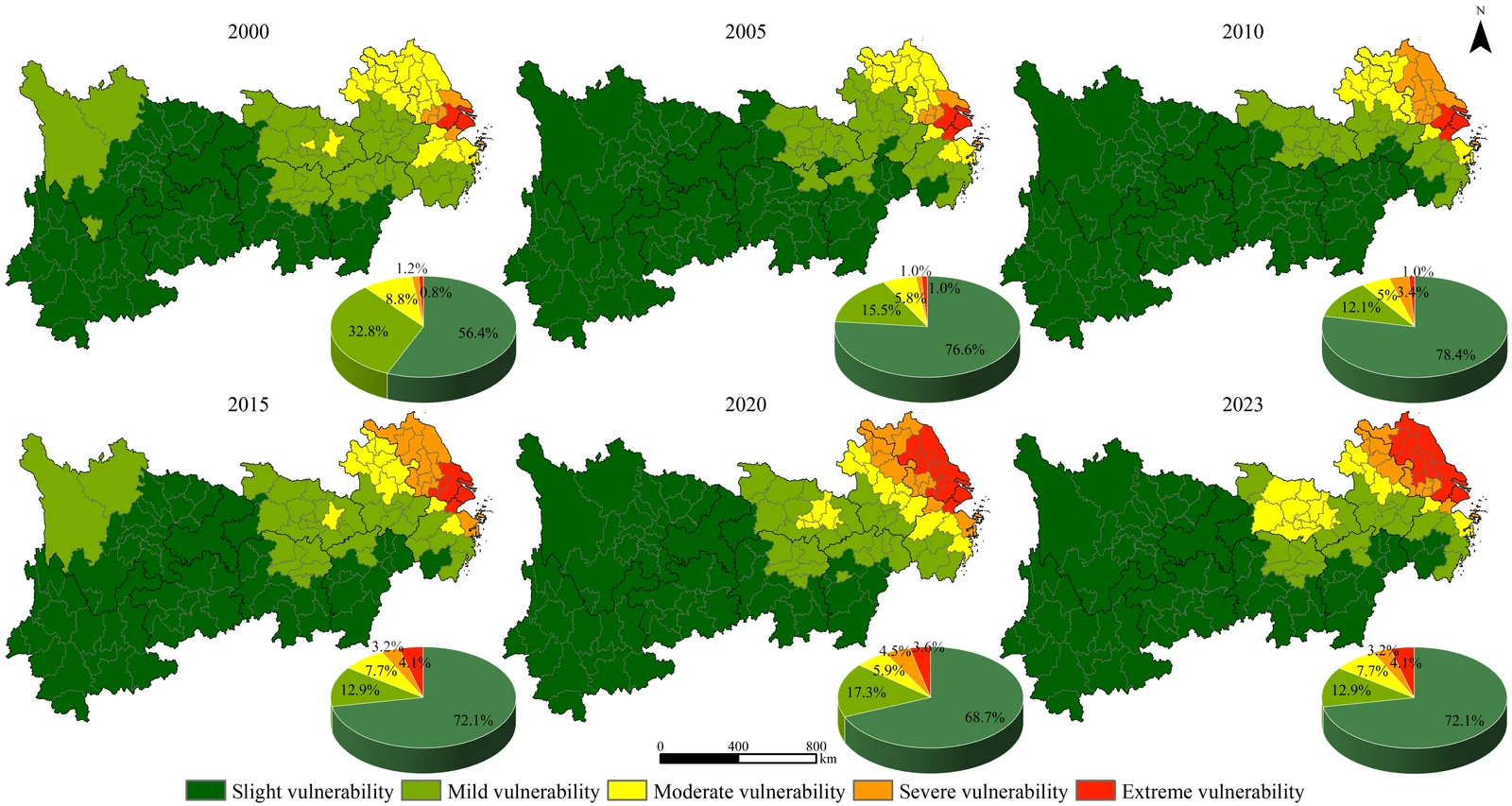
Spatiotemporal distribution of water system ecological vulnerability.

Spatially, ecological vulnerability of the water system in YREB exhibited a clustered distribution pattern that gradually decreased from the northeast toward the surrounding areas. Moderately vulnerable zones were primarily concentrated in the northeastern part of the Belt, including Zhejiang, Jiangsu, and Anhui, with scattered areas in central Hubei. The northeast region, located in the lower reaches of the Yangtze River, features flat terrain and a dense river network; however, natural factors such as seawater intrusion and sediment deposition weaken the self-purification capacity of the water system, resulting in generally moderate water quality. Severely and extremely vulnerable areas are mainly distributed in coastal regions, including Shanghai and Jiangsu. These coastal areas are highly developed economically, with dense industries and concentrated populations, leading to high water demand. The discharge of industrial wastewater and domestic sewage frequently causes water quality deterioration, resulting in high vulnerability. Areas with very slight and slight vulnerability accounted for most of the study area, with their combined proportion exceeding 80% in all years. Very slightly vulnerable zones were mainly distributed in the southern and western regions, such as Sichuan, Yunnan, Guizhou, Hunan, and Jiangxi, while slightly vulnerable zones were found in parts of the northwestern and central-eastern areas, such as Hubei and Anhui. The southern and western regions are dominated by mountainous and hilly terrain with high vegetation cover, providing strong water conservation capacity. In the central-eastern areas, the population is relatively smaller than in coastal regions, reducing pressure on water resources for production and domestic use, while lower industrial density limits the impact on water quality.

### 3.4 Spatiotemporal evolution of ecological vulnerability in the “Forest–Cropland–Water” system

From 2000 to 2023, the ecological vulnerability of the “Forest–Cropland–Water” system in YREB exhibited a fluctuating decline (Fig 6). From 2000 to 2010, vulnerability continuously decreased, with the areas of slight, mild, and moderate vulnerability expanding, accounting for increases of 13.58%, 7.81%, and 2.36%, respectively. The areas of severe and extreme vulnerability shrank, with their proportions decreasing by 17.89% and 5.86%, respectively. From 2010 to 2020, vulnerability gradually increased, with all vulnerability levels except slight vulnerability expanding; the proportion of slight vulnerability decreased by 16.68%. From 2020 to 2023, vulnerability markedly reduced, with the proportion of slight vulnerability rising by 11.59%, while the proportions of the other levels declined by 0.17%, 0.8%, 6.27%, and 4.34%, respectively.

**Fig 6:**
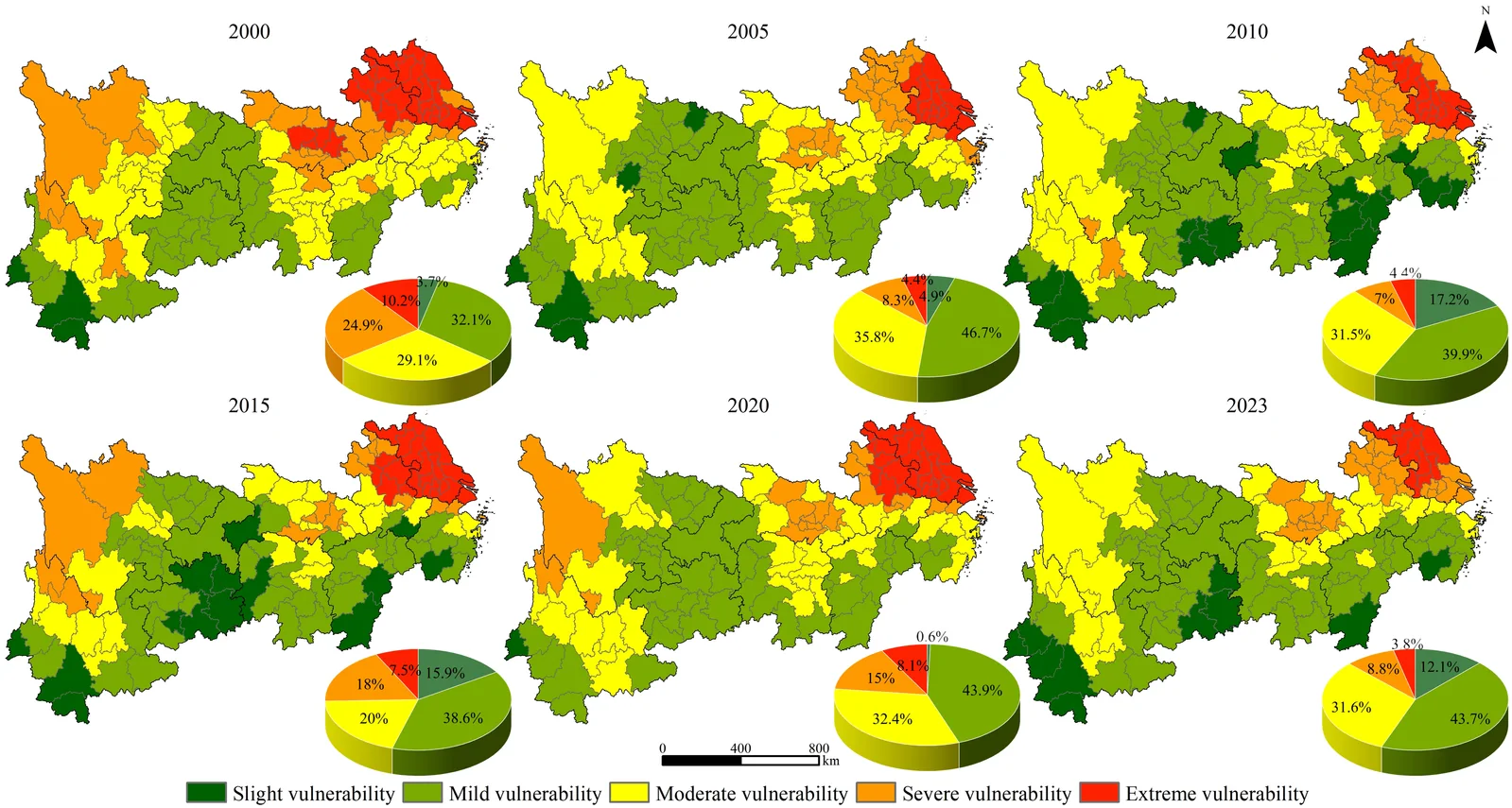
Spatiotemporal distribution of ecological vulnerability in the “Forest–Cropland–Water” system.

Spatially, the ecological vulnerability of the “Forest–Cropland–Water” system in YREB was high in the northeast and northwest, and low in the central and southern regions. Areas of moderate and severe vulnerability were primarily distributed in the northwest and central–eastern parts of the study area, while extreme vulnerability was concentrated in the northeast. Slight and light vulnerability areas were mostly located in the central and southern regions.

The provincial “Forest–Cropland–Water” system ecological vulnerability varied from 2000 to 2023 (Fig 7). Guizhou exhibited the lowest ecological vulnerability, with an average EVI of 0.36, while Jiangsu and Shanghai ranked the highest, with average EVIs of 0.68 and 0.67, respectively. The relatively low vulnerability in Guizhou can be attributed to its location on the Yunnan–Guizhou Plateau, where mountainous and hilly terrain predominates, forest coverage has long ranked among the highest nationwide, population density is low, and industrial development remains relatively weak. In contrast, Jiangsu and Shanghai, situated in the core area of the Yangtze River Delta, were characterized by intensive industrialization and urbanization. Human production and living activities exert substantial pressure on the regional ecological environment, and the priority given to economic growth has weakened the enforcement of environmental protection policies, resulting in increased vulnerability. In terms of temporal dynamics, the EVI across the 11 provinces of YREB showed an overall declining trend. Zhejiang experienced the greatest reduction, with a decrease rate of 27.8%, while Jiangsu exhibited the smallest decline, at only 0.07%. Zhejiang’s substantial improvement in ecological resilience can be primarily attributed to its industrial restructuring and upgrading, the establishment of China’s first cross-provincial ecological compensation mechanism, and the implementation of the “Five Waters Co-governance” policy, which collectively contributed to a marked decline in ecological vulnerability over the past two decades. Across the six time intervals, the year 2020 represented a turning point, when vulnerability levels rose across all provinces. This increase may be linked to post-pandemic economic recovery measures that stimulated industrial production resumption and triggered a rebound in energy demand, thereby intensifying pressures on the ecological system.

**Fig 7:**
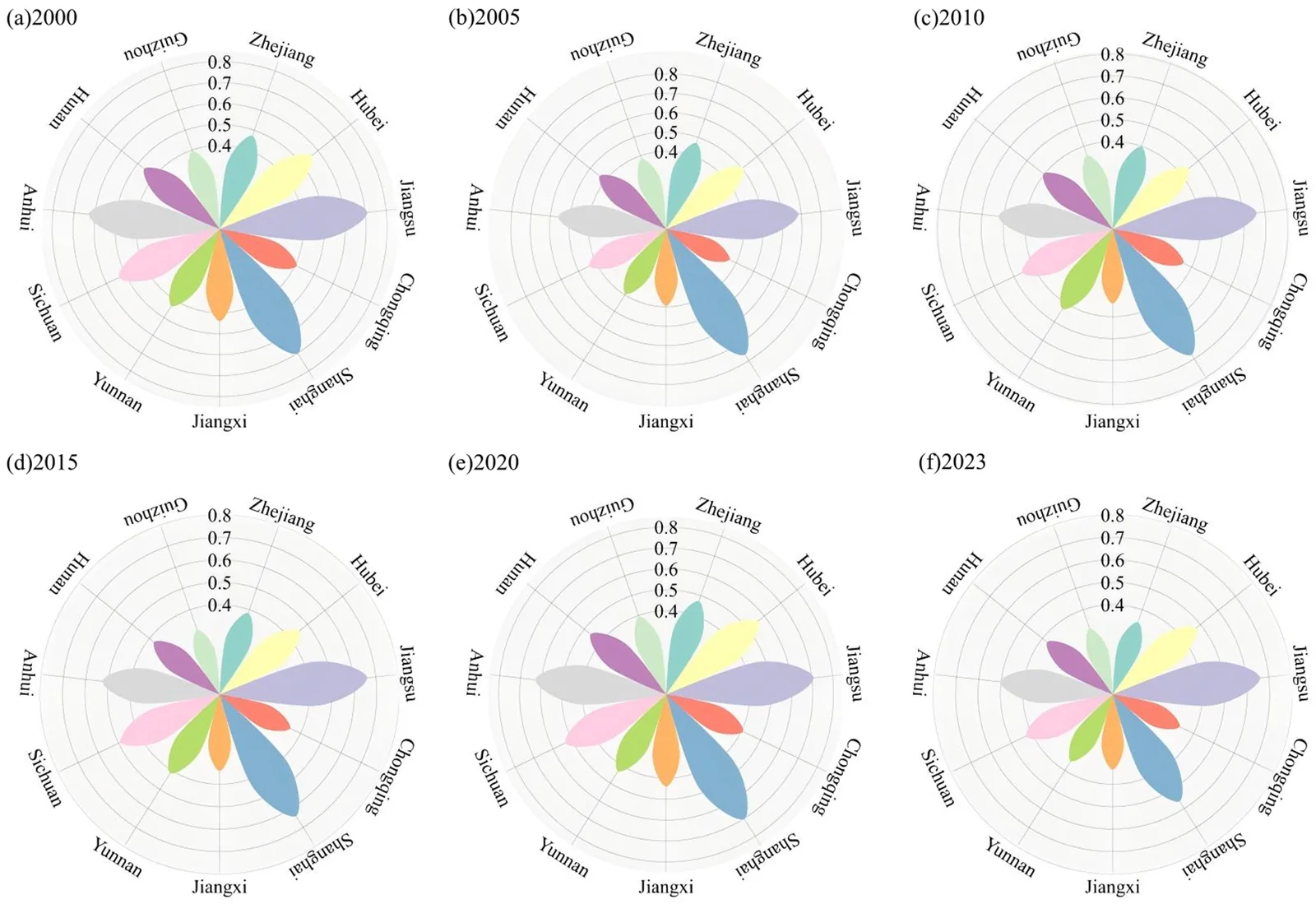
Ecological vulnerability of the “Forest–Cropland–Water” system in each province from 2000 to 2023.

### 3.5 Spatiotemporal evolution of the coupling coordination of the “Forest–Cropland–Water” system

According to the results of Moran’s I and significance test (Table 5), the P-values from 2000 to 2023 are all less than 0.001, indicating significant correlation at the 99.9% confidence level. In addition, all Z-scores are greater than 25, suggesting that the coupling coordination in YREB exhibited a pronounced spatial clustering pattern. Overall, Moran’s I showed a gradual decline, decreasing from 0.975 in 2000 to 0.855 in 2023, indicating that the spatial dependence of coupling coordination has weakened over time, with a tendency toward more dispersed distribution. Local spatial autocorrelation analysis at the prefecture and district levels further reveals (Fig 8) that coupling coordination was dominated by H–H and L–L clustering types. Specifically, the number of H–H clusters decreased from 48 in 2000–39 in 2023, while the number of L–L clusters increased from 46 to 48 over the same period. H–H clusters were mainly distributed in the northeastern part of the study area, indicating relatively high coordination of “Forest–Cropland–Water” system vulnerability. However, considering the spatial distribution of vulnerabilities across the three ecosystems, the northeastern region was characterized by generally high vulnerability, suggesting that the coordination occurs under conditions of high ecological fragility. In contrast, L–L clusters were primarily concentrated in the central-southern and western regions, reflecting lower levels of coordination. In these areas, mismatches exist among ecosystems; for instance, the water system is mostly classified as micro or slight vulnerability, whereas the forest and cropland systems are predominantly moderate to severe, thereby reducing the overall coordination level.

**Table 5:** Moran’s I statistics of the coupling coordination of the “Forest–Cropland–Water” system.

Year	Moran’s I	Z-scores	P-values
2000	0.975	29.438	<0.001
2005	0.920	27.844	<0.001
2010	0.910	27.552	<0.001
2015	0.851	25.733	<0.001
2020	0.908	27.452	<0.001
2023	0.855	25.889	<0.001

**Fig 8:**
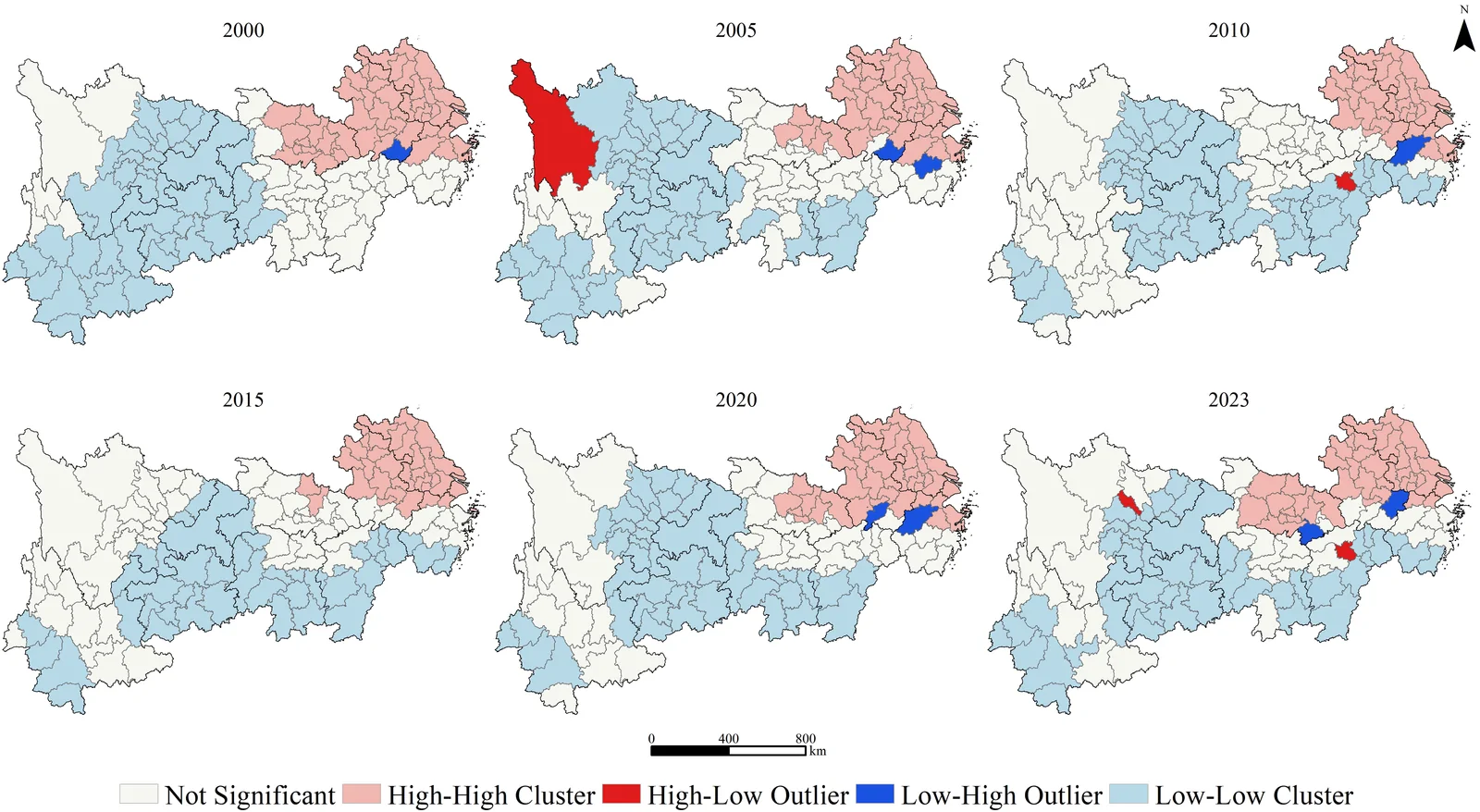
Spatial distribution of High/Low clusters of coupling coordination degree in YREB.

From 2000 to 2023, the coupling coordination degree of the “Forest–Cropland–Water” system in YREB exhibited an overall upward trend, despite fluctuations in certain years (Fig 9). At the provincial scale, the coordination degree in all provinces exceeded their 2000 levels by the end of the study period. Notably, Hubei and Anhui improved from a slight imbalance to slight coordination. In terms of the area proportions of different coupling–coordination types, the study area was predominantly characterized by the type “slight imbalance–moderate vulnerability”, which gradually shifted toward “slight imbalance–low vulnerability” and “slight coordination–moderate vulnerability” over time. From 2000 to 2023, the area proportions of these two categories showed fluctuating but overall upward trends, increasing by 29.84% and 23.57%, respectively.

**Fig 9:**
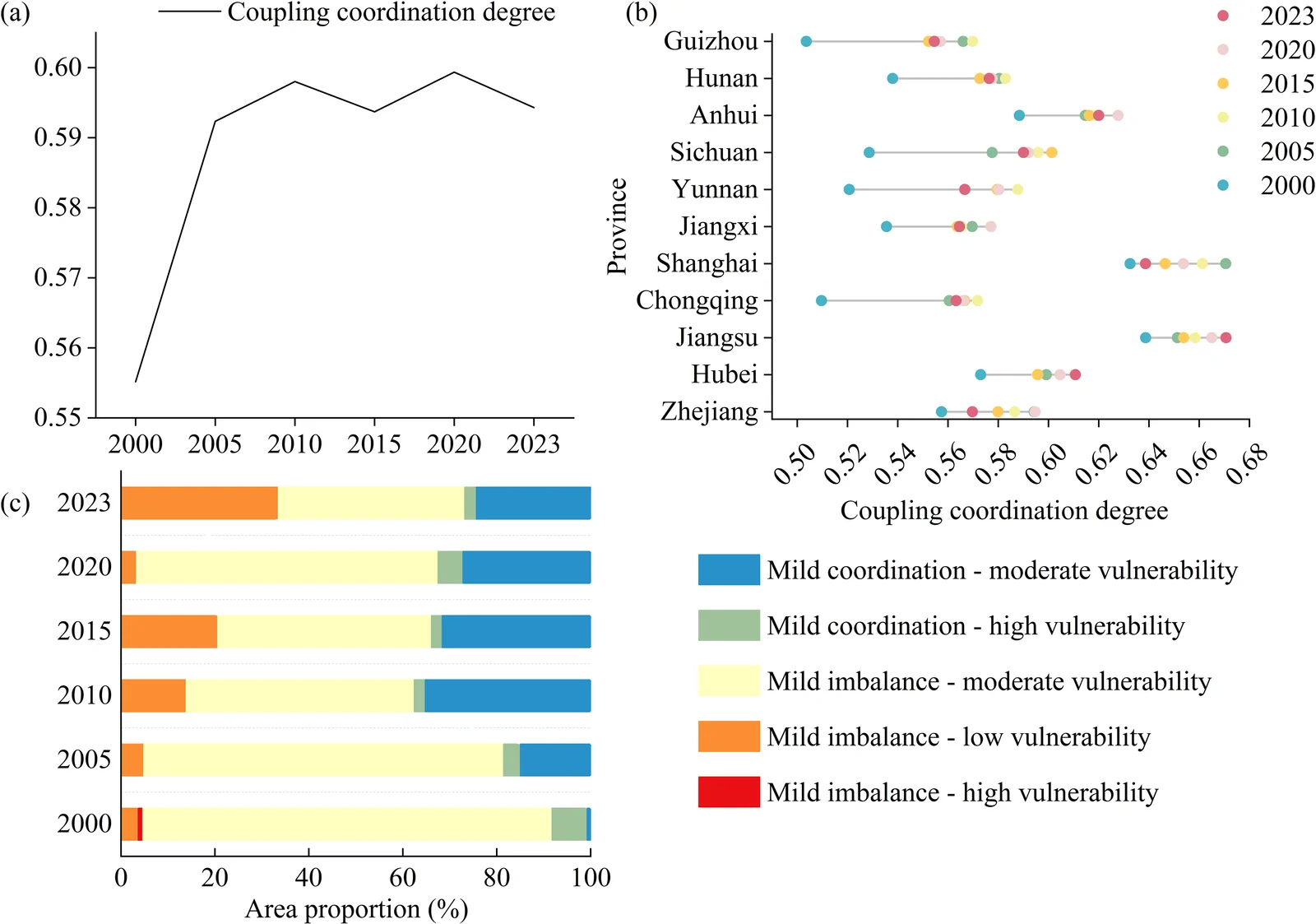
Coupling coordination degree and area proportion from 2000 to 2023: overall (a), by province (b), and area proportion (c).

From 2000 to 2023, the coupling coordination degree of the “Forest–Cropland–Water” system in YREB generally exhibited a spatial pattern of being higher in the central region and lower in the eastern and western regions (Fig 10). The coupling coordination values were relatively high in the western and northeastern regions, while lower in the central and southern regions. The type slight imbalance–high vulnerability indicates that the “Forest–Cropland–Water” system was imbalanced, with ecosystems experiencing either excessively high or excessively low values, and the overall vulnerability being relatively severe. This type was only sporadically distributed in 2000. Since 2005, overall vulnerability declined, the coupling coordination values increased, and the type transitioned into slight coordination–moderate vulnerability. The slight coordination–high vulnerability type suggests that although the coupling index of the “Forest–Cropland–Water” system was relatively balanced, vulnerability level remained severe. This type was mainly distributed in the northeastern Yangtze River Delta, including Jiangsu and Shanghai. With the advancement of Yangtze River Delta integration and the implementation of policies such as the Yangtze River Delta Regional Environmental Cooperation Declaration, ecological civilization construction was promoted, gradually establishing a comprehensive regional environmental protection system, which reduced ecological vulnerability. The slight imbalance–low vulnerability type indicates relatively low vulnerability and clear ecological advantages, but weaker synchronization in the coordination of the “Forest–Cropland–Water” system, with mutual constraints among ecosystems. Its distribution was relatively scattered, initially concentrated in parts of Yunnan. With improvements in economic development and increased investment in ecological protection, vulnerability levels declined year by year. Except for 2020, when vulnerability temporarily rose and the spatial extent shrank, its distribution area gradually expanded to Guizhou, Chongqing, Jiangxi, and Zhejiang. The slight imbalance–moderate vulnerability type reflects moderate levels of both coupling coordination and vulnerability. This category accounted for most of the study area, forming large contiguous clusters in the central region. Over time, its spatial extent gradually decreased and was progressively replaced by slight coordination, moderate vulnerability, and slight imbalance, low vulnerability. The slight coordination–moderate vulnerability type indicates that the regional “Forest–Cropland–Water” system exhibited a trend of synchronized coordination with moderate vulnerability. It was mainly distributed across the eastern and western parts of the study area, showing a significant expansion from 2000 to 2010, followed by a gradual contraction between 2010 and 2023.

**Fig 10:**
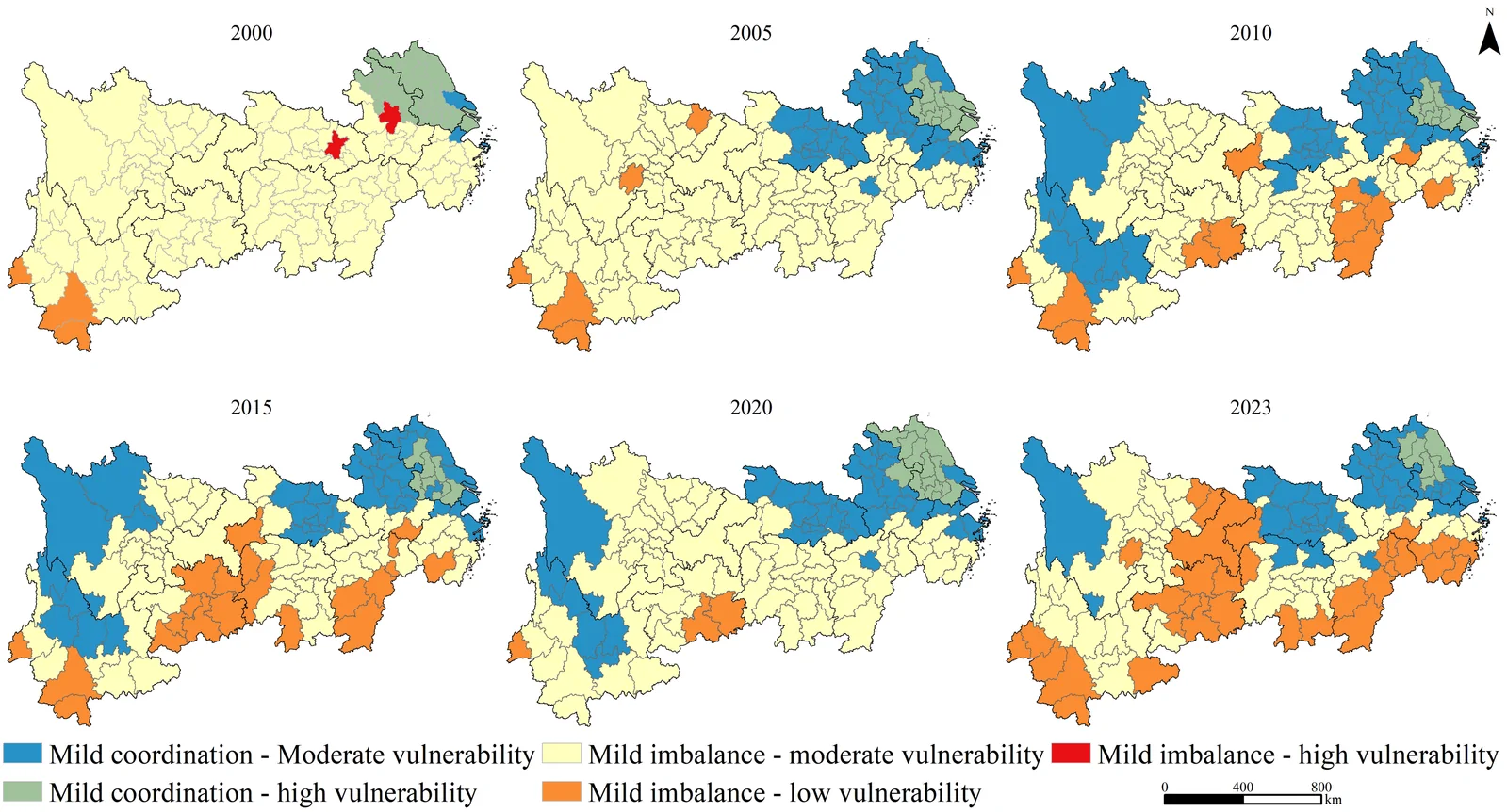
Spatiotemporal distribution of coupling coordination in the “Forest–Cropland–Water” system.

### 3.6 Driving factor analysis of the Forest–Cropland–Water system coupling

Based on the geodetector q-values, the explanatory power of the six selected indicators for the coupling coordination degree of the “Forest–Cropland–Water” system in the YREB exhibited distinct spatiotemporal patterns over 2000–2023, the results are presented in Fig 11. LUI emerged as the consistently strongest driver, with its q-value rising from 0.280 in 2000 to 0.389 in 2023, indicating a progressively reinforced influence of anthropogenic land–use restructuring on the coordination among the three ecosystems. AOD also showed substantial explanatory capacity, particularly peaking at 0.370 in 2015, which underscores the role of atmospheric particulate loading in modulating vegetation productivity and hydrological processes, thereby affecting overall system balance. In contrast, HTD displayed a marked decreasing trend in q-values, from 0.406 in 2000 to 0.078 in 2023, suggesting that the spatial dominance of heat extremes as a coordinating factor has considerably weakened over time, possibly due to improved ecological resilience or shifting climatic patterns. Meanwhile, NL, ERD, and TWS maintained persistently low q-values (mostly below 0.15) throughout the study period, implying their limited independent contributions to the spatial heterogeneity of coupling coordination. Notably, despite TWS being a direct measure of water availability, its weak explanatory power in the geodetector framework suggests that water-related impacts on coordination are more effectively mediated through other pathways. Overall, these findings highlight that land use intensity and atmospheric aerosol loading are the predominant spatial drivers, while the role of extreme temperature events has diminished, and other selected factors play only subsidiary roles in shaping the coordinated evolution of the “Forest–Cropland–Water” system within the study region.

**Fig 11:**
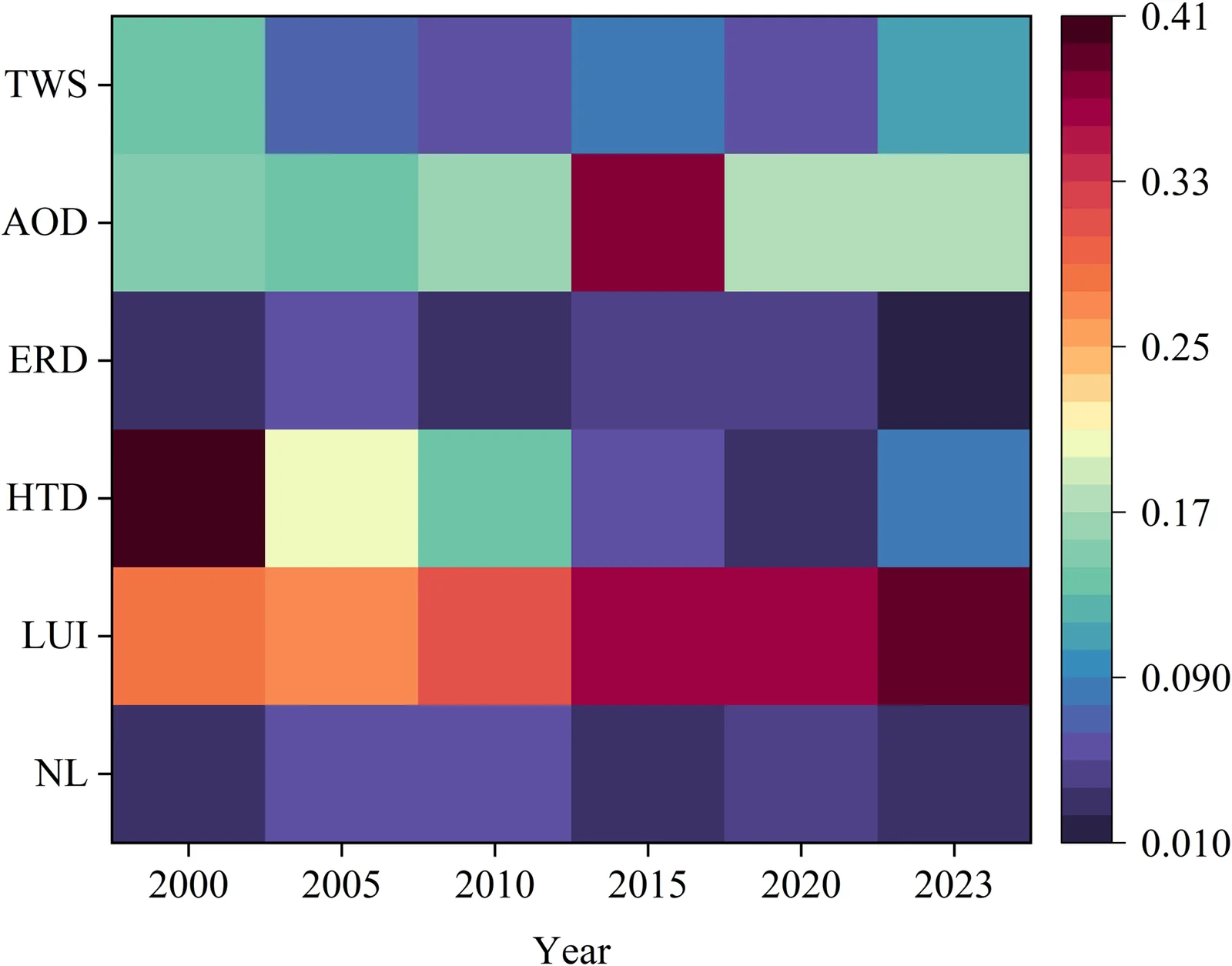
Driving strength of each indicator on coupling coordination.

## 4 Discussion

### 4.1 Spatiotemporal evolution patterns of ecological vulnerability in the “Forest–Cropland–Water” system of YREB

From 2000 to 2023, the ecological vulnerability of the “Forest–Cropland–Water” system in YREB exhibited a “decrease-increase-decrease” trajectory. This evolution reflected the combined effects of policy regulation, economic development, climate change, and ecological responses. This finding is largely consistent with the results proposed by Luo et al. [81], who suggested that the ecological vulnerability of the YREB initially increased and then decreased. Spatially, the comprehensive ecological vulnerability of the three systems exhibited a distribution pattern of being higher in the northeast and lower in the south, which is broadly consistent with the findings of Wei [82] et al., who reported a nort-high and south-low spatial pattern of ecological sensitivity in the YREB. This correspondence reflects a relatively high degree of consistency in the ecological and environmental conditions across the various regions of the belt.

Initially, the vulnerability declined during 2000–2010 primarily due to the sustained implementation of ecological restoration projects. The full-scale launch of the Grain-for-Green Program in 2002, along with the implementation of forest protection policies, enhanced forest coverage and improved soil and water conservation capabilities. The 10th Five-Year Plan for National Economic and Social Development (2006) proposed the protection of cropland red lines, improving cropland planting environments, standardizing cropland compensation and replacement, and enhancing cropland quality. Meanwhile, hydropower development and water area expansion optimized regional water resource allocation, while the conversion of cropland to lakes increased wetland areas and enhanced flood regulation capacity.

The vulnerability again increased during 2010–2020 due to multiple conflicting factors. Rapid urbanization expanded construction land, encroaching on forest and wetland areas and exacerbating soil erosion. Climate change has led to frequent extreme weather events, weakening ecosystem resilience, while imbalances in water resource utilization further exacerbated the supply-demand contradiction. However, vulnerability declined again after 2020 due to systematic ecological and environmental governance, including the delineation of “three zones and three lines” in national land-use planning to optimize land-use structure. The Yangtze River Ecological Protection Strategy, which promoted overall water quality improvement, also enhanced forest carbon sink capacity and strengthened ecosystem stability; while the ecological transformation of agriculture reduced soil pollution at its source.

In summary, the first decade yielded major policy dividends from ecological restoration efforts, while the middle decade exhibited increased ecosystem vulnerability due to urbanization and pollution pressures. After 2020, the ecological environment gradually improved through refined governance measures.

### 4.2 Ecological vulnerability coupling and coordination of “Forest–Cropland–Water” system in YREB

From 2000 to 2023, the coupling coordination degree of the “Forest–Cropland–Water” system in the YREB exhibited a fluctuating upward trend, while the degree of spatial agglomeration gradually weakened (Moran’s I declined from 0.975 to 0.855), and the coupling type transitioned from mild imbalance-moderate vulnerability to mild coordination–moderate vulnerability. This upward trend is consistent with the findings of Lv et al [83]. on the coupling coordination of the water-energy-food nexus system; however, the weakening of spatial agglomeration differs from the results of Liu et al. [25], who found a continuously strengthening spatial differentiation of energy–economy–ecology coupling coordination. This discrepancy primarily stems from the fundamental difference in research subjects: Liu et al. focused on cross-domain coupling between socio‑economic systems and the ecological environment, where significant regional disparities in economic development levels led to persistently increasing spatial differentiation; whereas the present study concentrates on the coupling among three systems within the natural ecosystem, where ecological restoration projects are characterized by region‑wide coverage, and their ecological benefits tend to become homogenized through processes such as hydrological connectivity and atmospheric transport, thereby weakening spatial agglomeration. Furthermore, we found that the vulnerabilities of the three systems exhibited synchronous changes during most of the study period, indicating that ecological restoration measures, through multidirectional transmission involving hydrological regulation, soil conservation, and climatic buffering, have achieved synergistic improvement across the three systems. In summary, this study reveals the distinct evolution patterns of coupling within natural ecosystems as opposed to socio‑ecological cross‑domain coupling, providing a scientific basis from a system coordination perspective for the integrated protection of “mountains, waters, forests, farmlands, lakes, grasslands, and deserts”: management policies should shift from single‑system governance to multi-system synergistic optimization, with particular emphasis on matching vulnerabilities among systems to achieve balanced and coordinated development of the three systems.

### 4.3 Drivers of the coupling coordination degree of the “Forest–Cropland–Water” system in YREB

The driving-factor results revealed that, over the period from 2000 to 2023, land use intensity exerted the strongest influence on the coupling coordination degree of the “Forest–Cropland–Water” system in the YREB. This finding is broadly consistent with the results of Bi et al [84], who identified land use intensity as the primary determinant of the coupling coordination degree between urbanization and water-related ecosystem services, with pronounced spatial heterogeneity in the driving forces. This strongly corroborates the critical role of socio-economic factors in shaping system coordination within the YREB [85]. Meanwhile, AOD also exhibited substantial spatial explanatory power, which may be attributed to its modulation of regional water-heat balances through alterations in radiation budgets and physical processes, thereby affecting vegetation growth and hydrological cycling, and consequently influencing system coupling coordination. Existing studies have revealed that AOD is significantly negatively correlated with NDVI and precipitation across the Yangtze River Basin [86], and that AOD constitutes a primary driver of leaf area index changes in China [87]—findings that further reinforce the conclusions of this study. In summary, the driving-factor results of this study are highly consistent with the broader literature on the YREB in confirming the primacy of anthropogenic land use intensity as a driver, while the significant role of AOD aligns with accumulating evidence of aerosol impacts on vegetation and hydrological processes. Based on the driving-factor analysis results, this study proposes the following future development recommendations. First, given that LUI is the strongest driver with a persistently increasing trend, strict controls should be imposed on the disorderly expansion of built-up land in the YREB, alongside optimization of territorial spatial development patterns and promotion of intensive and efficient land-use practices, so as to alleviate anthropogenic pressures on forests, croplands, and water systems [62]. Second, given the substantial spatial explanatory power of AOD, it is advisable to strengthen regional joint prevention and control mechanisms for air pollution to reduce the negative impacts of aerosol emissions on vegetation productivity and hydrological cycling. Moreover, the marked decline in the explanatory power of HTD indicates that ecological restoration projects have been effective in enhancing system climate resilience; therefore, systematic ecological initiatives such as the Grain-for-Green Program and the Yangtze River Protection Initiative should be continuously advanced to consolidate their positive role in buffering against climate extremes [88]. Finally, the limited explanatory power of NL, ERD, and TWS suggests that their influences are primarily mediated through other pathways such as land use or vegetation cover; consequently, a multi-system synergistic governance framework is recommended, in which water resource management is integrated with land-use planning and air pollution control, to achieve balanced coordination and sustainable development of the “Forest–Cropland–Water” system [89].

### 4.4 Limitations and future directions

This study innovatively develops a multi-system coupling evaluation framework by employing a combined G1–CRITIC–game theory combination weighting to construct a regional “Forest–Cropland–Water” composite system ecological vulnerability assessment model and coupling-coordination typology. At the provincial scale, we systematically analyze the coupling mechanisms among the three ecosystems and apply spatial autocorrelation techniques to reveal their patterns of spatial clustering. Further, a Geodetector method is used to quantitatively identify the key drivers of the spatiotemporal evolution of system coupling coordination. However, certain limitations remain. First, some statistical yearbook data for the study period were unavailable and were replaced with figures from adjacent years, which may have affected cross-sectional comparability, additionally, because some data are qualitative and lack temporal variation, this may result in a lack of significant temporal change. Second, our analysis units are provinces and prefecture-level cities, with relatively few investigations at finer, more localized scales. Future work could undertake more detailed analyses at the county level.

## 5 Conclusion

(1) From 2000 to 2023, the ecological vulnerability of all ecosystems and the overall ecological vulnerability of YREB showed a fluctuating downward trend. Spatially, the ecological vulnerability of cropland decreased in a stepwise manner from west to east, while the ecological vulnerability of other ecosystems and the overall ecological vulnerability exhibited a distribution pattern of higher values in the north and lower values in the south.
(2) At the provincial level, Guizhou had the lowest ecological vulnerability over the years, with an average EVI of 0.36; while Jiangsu had the most severe ecological vulnerability, with an average EVI of 0.68. In terms of trends, the EVI of all 11 provinces showed a decreasing trend, with Zhejiang experiencing the largest decrease, at a rate of 27.8%.
(3) From 2000 to 2023, the coupling coordination degree of the “Forest–Cropland–Water” system in YREB fluctuated upward, while the spatial aggregation degree decreased year by year. The overall coupling coordination type in the study area is primarily mild imbalance-moderate vulnerability, gradually shifting toward mild coordination-moderate vulnerability and mild imbalance-low vulnerability over time. Among the provinces, the coupling coordination degree of all provinces exceeded that of 2000 by the end of the study period.
(4) Overall, over the period from 2000 to 2023, anthropogenic land-use alteration and atmospheric aerosol loading are the predominant spatial determinants, whereas climatic extremes and water-storage indicators exert secondary influences on the coordinated evolution of the system.
(5) This study innovatively developed a comprehensive assessment framework that integrates subjective and objective weighting, multi-system coupling coordination analysis, and identification of key driving factors, enabling a transition from “static assessment of a single system” to “dynamic diagnosis of multi-system coupling.” This framework provides a scientific and quantitative analytical template for the evaluation of regional composite ecosystems.

## References

[1] Zhu Q, Wang Y, Zhou W, Zhou L, Yu D, Qi L. Spatiotemporal changes and driving factors of ecological vulnerability in Northeast China forest belt. Shengtaixue Zazhi. 2021;40:3474–82. doi: 10.13292/J.1000-4890.202111.021

[2] Bin F, Li X, Zhou X, Zhang Z. Diagnosis of cultivated land use system vulnerability and identification of influencing factors: A case study of northern Jiangsu. China Land Science. 2024;38:79–90.

[3] Xu W, Zhang X, Cui Y, Tian T, Lin L, Liu Y. Study on Comprehensive Evaluation of Urban Water Resource Vulnerability. Sustainability. 2022;14(7):4149. doi: 10.3390/su14074149

[4] Liu X, Wang C, Wang Y, Zou C, Hou P. Evaluation on the implementation effect of ecological protection and restoration project of Mountain-Water-Forest-Cropland-Lake-Grass system in Qilian mountains, Qinghai Province. Acta Ecologica Sinica. 2024;44:5960–73. doi: 10.20103/j.stxb.202310052136

[5] Chen Y, Zhang Y, Zhang X, Zhang Y. The institutional construction of territorial spatial governance for “integrated mountains, rivers, forests, fields, lakes, grasses and deserts” in China. Journal of Natural Resources. 2025;40(5):1174. doi: 10.31497/zrzyxb.20250503

[6] Liu H, Xiong K, Yu Y, Li T, Qing Y, Wang Z, et al. A Review of Forest Ecosystem Vulnerability and Resilience: Implications for the Rocky Desertification Control. Sustainability. 2021;13(21):11849. doi: 10.3390/su132111849

[7] Sauke F, Fischer R, Rode M. A review on modelling forest biogeochemistry and the coupled forest – soil interactions in a changing world. Environmental Modelling & Software. 2025;187:106381. doi: 10.1016/j.envsoft.2025.106381

[8] Thakur S, Dhyani R, Negi VS, Patley MK, Rawal RS, Bhatt ID, et al. Spatial forest vulnerability profile of major forest types in Indian Western Himalaya. Forest Ecology and Management. 2021;497:119527. doi: 10.1016/j.foreco.2021.119527

[9] Liao Y, Xie E, Chen W, Yao D, Lei M, Dang Y. Comprehensive zoning of cultivated land quality improvement based on synergistic production characteristics and ecological stress in China. Transactions of the Chinese Society of Agricultural Engineering. 2025;41:208–17.

[10] Li L, Jiang P, Liu W, Sun Y, Dang Z. Increasing Spatial Mismatch of Cropland-Grain Production-Population in China over the Past Two Decades. Land. 2022;11(10):1685. doi: 10.3390/land11101685

[11] Wu L, Qian C, Shen Y, Sun D. Assessment and Factor Diagnosis of Water Resource Vulnerability in Arid Inland River Basin: A Case Study of Shule River Basin, China. Sustainability. 2023;15(11):9052. doi: 10.3390/su15119052

[12] Chen W, Chen Y, Feng Y. Assessment and Prediction of Water Resources Vulnerability Based on a NRS-RF Model: A Case Study of the Song-Liao River Basin, China. Entropy (Basel). 2021;23(7):882. doi: 10.3390/e23070882 34356423 PMC8305098

[13] Li S, Liu Y, Shao Y, Wang X. Impact of Climate Change on Crop-cropland Coupling Relationship: A Case Study of the Loess Plateau in China. Chin Geogr Sci. 2025;35:92–110. doi: 10.1007/s11769-025-1487-4

[14] Ji J, Chen J, Ding T, Li Y. Coupling coordination between urban flood resilience and ecosystem services in the Yangtze River Delta urban agglomerations. Acta Ecologica Sinica. 2024;44:2772–85. doi: 10.20103/j.stxb.202304070696

[15] Hou K, Tao W, Wang L, Li X. Study on hierarchical transformation mechanisms of regional ecological vulnerability and its applicability. Ecological Indicators. 2020;114:106343. doi: 10.1016/j.ecolind.2020.106343

[16] Zhang X, Liu K, Wang S, Wu T, Li X, Wang J, et al. Spatiotemporal evolution of ecological vulnerability in the Yellow River Basin under ecological restoration initiatives. Ecological Indicators. 2022;135:108586. doi: 10.1016/j.ecolind.2022.108586

[17] Hou K, Tao W, He D, Li X. A new perspective on ecological vulnerability and its transformation mechanisms. Ecosyst Health Sustain. 2022;8(1). doi: 10.1080/20964129.2022.2115403

[18] Zhang R, Hu C, Lim H, Zhang Y. Spatiotemporal analysis and influencing factors of ecological vulnerability in the Tuojiang River Basin, China. Geocarto International. 2025;40(1). doi: 10.1080/10106049.2025.2453020

[19] Meru LB, Pandey R. Climate change ecological vulnerability and hotspot analysis of himalayan forests in North-Eastern region, India. Environmental and Sustainability Indicators. 2024;24:100472. doi: 10.1016/j.indic.2024.100472

[20] Li Y, Fang B, Zhang Z. Evolution and optimization of cropland use vulnerability in urbanization process: a case of the Huaihai economic zone. Resources and Environment in the Yangtze Basin. 2024;33:2410–24. doi: 10.11870/cjlyzyyhj202411008

[21] Chen Y, Feng Y. Assessment and prediction of water resources vulnerability in river basin based on RS-SVR model: a case study of the Yellow River basin. Resources and Environment in the Yangtze Basin. 2020;29:137–49.

[22] Zhao C, Yang N, Wang Z, Liu S, Dong X, Xin W. Benefit assessment of soil and water conservation from cropland to forest in hilly Loess Plateau at Qinghai. Springerplus. 2013;2(Suppl 1):S7. doi: 10.1186/2193-1801-2-S1-S7 24701390 PMC3973411

[23] Xiang Y, Shao W, Dai Z, Zhang Y, Ding S. Regional Differences and Convergence of Urban Water Use Efficiency in the Yangtze River Economic Belt. Water. 2023;15(13):2440. doi: 10.3390/w15132440

[24] Fan X, Zhou Y. Coupling coordination of land use and economic-social-environmental in the Yangtze River economic belt. Research of Soil and Water Conservation. 2023;30:370–8. doi: 10.13869/j.cnki.rswc.2023.05.014

[25] Liu J, Tian Y, Huang K, Yi T. Spatial-temporal differentiation of the coupling coordinated development of regional energy-economy-ecology system: A case study of the Yangtze River Economic Belt. Ecological Indicators. 2021;124:107394. doi: 10.1016/j.ecolind.2021.107394

[26] Liu H, Zhang X, Tao S, Xiao X, Wu K, Xia J. A Coevolution Model of the Coupled Society—Water Resources—Environment Systems: An Application in a Case Study in the Yangtze River Economic Belt, China. Water. 2022;14(15):2449. doi: 10.3390/w14152449

[27] Ye C, Pi J, Chen H. Coupling Coordination Development of the Logistics Industry, New Urbanization and the Ecological Environment in the Yangtze River Economic Belt. Sustainability. 2022;14(9):5298. doi: 10.3390/su14095298

[28] Jiang Z, Zhou Y, Gao S, Dong Z, Wang Y, Duan Z, et al. Stronger effects of accumulated soil moisture deficit on gross primary productivity and light use efficiency than lagged soil moisture deficit for cropland and forest. Agricultural and Forest Meteorology. 2025;361:110317. doi: 10.1016/j.agrformet.2024.110317

[29] Bonsch M, Popp A, Biewald A, Rolinski S, Schmitz C, Weindl I, et al. Environmental flow provision: Implications for agricultural water and land-use at the global scale. Global Environmental Change. 2015;30:113–32. doi: 10.1016/j.gloenvcha.2014.10.015

[30] Li M, Wang G, Sun A, Wang Y, Li F, Liang S. Monitoring Grassland Variation in a Typical Area of the Qinghai Lake Basin Using 30 m Annual Maximum NDVI Data. Remote Sensing. 2024;16(7):1222. doi: 10.3390/rs16071222

[31] Cao E. Spatiotemporal changes of ecological vulnerability in Ziwuling area based on the VSD model. Lanzhou University. 2021.

[32] Dunkerley D. Rainfall intensity in short events: Evaluating the “I30 is equal to twice the rainfall depth” approach advised for use with the Universal Soil Loss Equation by Wischmeier & Smith (1978). Catena. 2021;207:105659. doi: 10.1016/j.catena.2021.105659

[33] Tian P, Jia T, Ping Y, Xu Y, Wang Z, Liu M. Spatial-Temporal Differentiation and Attribution of Soil Erosion in Northwestern Hubei Based on RUSLE and Geographic Detector. Tropical Geography. 2023;43:2216–28. doi: 10.13284/j.cnki.rddl.003728

[34] Gao J, Shi Y, Zhang H, Chen X, Zhang W, Shen W. China regional 250m normalized difference vegetation index data set (2000-2023). National Tibetan Plateau / Third Pole Environment Data Center. doi: 10.11888/Terre.tpdc.300328

[35] Steve R, Zhao M. MODIS/Terra Net Primary Production Gap-Filled Yearly L4 Global 500m SIN Grid V061 | NASA Earthdata. Earth Science Data Systems, NASA. 2025. https://www.earthdata.nasa.gov/data/catalog/lpcloud-mod17a3hgf-061

[36] Xu X. Annual NDVI and EVI 1-km datasets for China. Resource and Environmental Science Data Platform. 2018. doi: 10.12078/201806060

[37] Yang J, Huang X. The 30 m annual land cover dataset and its dynamics in China from 1990 to 2019. Earth Syst Sci Data. 2021;13(8):3907–25. doi: 10.5194/essd-13-3907-2021

[38] Chen Y, Yang H, Yang Z, Yang Q, Liu W, Huang G, et al. Enhancing high-resolution forest stand mean height mapping in China through an individual tree-based approach with close-range lidar data. Earth Syst Sci Data. 2024;16(11):5267–85. doi: 10.5194/essd-16-5267-2024

[39] Cai Y, Xu X, Zhu P, Nie S, Wang C, Xiong Y, et al. Unveiling spatiotemporal tree cover patterns in China: The first 30 m annual tree cover mapping from 1985 to 2023. ISPRS Journal of Photogrammetry and Remote Sensing. 2024;216: 240–58. doi: 10.1016/j.isprsjprs.2024.08.001

[40] Gupta S, Borrelli P, Panagos P, Alewell C. An advanced global soil erodibility (K) assessment including the effects of saturated hydraulic conductivity. Sci Total Environ. 2024;908:168249. doi: 10.1016/j.scitotenv.2023.168249 37918722

[41] Guo K, Ji Q, Zhang D. A dataset to measure global climate physical risk. Data Brief. 2024;54:110502. doi: 10.1016/j.dib.2024.110502 38774240 PMC11106815

[42] Shangguan W, Dai Y, Liu B, Zhu A, Duan Q, Wu L, et al. A China data set of soil properties for land surface modeling. J Adv Model Earth Syst. 2013;5:212–24. doi: 10.1002/jame.20026

[43] Hengl T, Mendes de Jesus J, Heuvelink GBM, Ruiperez Gonzalez M, Kilibarda M, Blagotić A, et al. SoilGrids250m: Global gridded soil information based on machine learning. PLoS One. 2017;12(2):e0169748. doi: 10.1371/journal.pone.0169748 28207752 PMC5313206

[44] Nyberg B. Surface Area of River and Lakes (SARL). Zenodo. 2023. doi: 10.5281/ZENODO.6895820

[45] Peng S, Gang C, Cao Y, Chen Y. Assessment of climate change trends over the Loess Plateau in China from 1901 to 2100. Intl Journal of Climatology. 2017;38(5):2250–64. doi: 10.1002/joc.5331

[46] Peng S, Ding Y, Wen Z, Chen Y, Cao Y, Ren J. Spatiotemporal change and trend analysis of potential evapotranspiration over the Loess Plateau of China during 2011–2100. Agricultural and Forest Meteorology. 2017;233:183–94. doi: 10.1016/j.agrformet.2016.11.129

[47] Peng S. 1-km monthly precipitation dataset for China (1901-2025). National Tibetan Plateau / Third Pole Environment Data Center. doi: 10.5281/zenodo.3114194

[48] Peng S. 1-km monthly mean temperature dataset for china (1901-2025). National Tibetan Plateau / Third Pole Environment Data Center. 2020. doi: 10.11888/Meteoro.tpdc.270961

[49] Zhang D, Peng J. A dataset of 1km grid drainage density in China. 2024. https://cstr.cn/31253.11.sciencedb.16001

[50] Peng J, Zhang D, Li Y, Zhang X. A dataset of 1 km grid drainage density in China. CSD. 2024;9:fpage-lpage. doi: 10.11922/11-6035.csd.2022.0084.zh

[51] Tian Y, Cheng KM, Zhang Z, Zhang T, Feng J, Ren Z, et al. An Extended VIIRS-like Artificial Nighttime Light Data Reconstruction (1986-2024). Sci Data. 2026;13(1):233. doi: 10.1038/s41597-026-06549-0 41577719 PMC12902030

[52] Wu B. Land Use Intensity and Landscape Ecological Risk Datasets for China (1992–2022). 2026. doi: 10.5281/zenodo.18938572

[53] Chen W, Xiong Y, Zhong M, Yang Z, Shum CK, Li W, et al. Twenty-Year Spatiotemporal Variations of TWS over Mainland China Observed by GRACE and GRACE Follow-On Satellites. Atmosphere. 2023;14(12):1717. doi: 10.3390/atmos14121717

[54] Bai K, Ke L. LGHAP v2: Global monthly mean 1-km gap-free AOD grids (2000-2024). 2025. doi: 10.5281/zenodo.15879684

[55] Zhang Y, Xiong K, Chen Y, Bai X. Spatiotemporal changes and driving factors of ecological vulnerability in karst World Heritage sites based on SRP and geodetector: a case study of Shibing and Libo-Huanjiang karst. npj Herit Sci. 2025;13(1). doi: 10.1038/s40494-025-01584-3

[56] Roshani, Sajjad H, Rahaman MH, Masroor M, Sharma Y, Sharma A, et al. Vulnerability assessment of forest ecosystem based on exposure, sensitivity and adaptive capacity in the Valmiki Tiger Reserve, India: A geospatial analysis. Ecological Informatics. 2024;80:102494. doi: 10.1016/j.ecoinf.2024.102494

[57] Wu S, Zeng G, Sun J, Liu X, Li X, Zeng Q, et al. Assessment of the Spatiotemporal Evolution Characteristics and Driving Factors of Ecological Vulnerability in the Hubei Section of the Yangtze River Economic Belt. Land. 2025;14(5):996. doi: 10.3390/land14050996

[58] Wang Z, Chen W, Piao J, Cai Q, Chen S, Xue X, et al. Synergistic effects of high atmospheric and soil dryness on record-breaking decreases in vegetation productivity over Southwest China in 2023. NPJ Clim Atmos Sci. 2025;8:6. doi: 10.1038/s41612-025-00895-3

[59] Li W, Migliavacca M, Forkel M, Denissen JMC, Reichstein M, Yang H, et al. Widespread increasing vegetation sensitivity to soil moisture. Nat Commun. 2022;13(1):3959. doi: 10.1038/s41467-022-31667-9 35803919 PMC9270344

[60] Qiu M, Fu M, Zhang Z, Fu S, Yuan C. Assessing the ecological risk of croplands in loess drylands by combining environmental disturbance with ecosystem vulnerability. J Environ Manage. 2023;347:119231. doi: 10.1016/j.jenvman.2023.119231 37804628

[61] Fang W, Yang Z, Liu Z, Hatab AA. Green recovery of cropland carrying capacity in developed regions: empirical evidence from Guangdong, China. Econ Chang Restruct. 2023;56:2405–36. doi: 10.1007/s10644-023-09519-3

[62] Fu Z, Ciais P, Wigneron J-P, Gentine P, Feldman AF, Makowski D, et al. Global critical soil moisture thresholds of plant water stress. Nat Commun. 2024;15(1):4826. doi: 10.1038/s41467-024-49244-7 38844502 PMC11156669

[63] Gezici K, Şengül S, Kesgin E. Assessment of soil erosion risk in the mountainous region of northeastern Türkiye based on the RUSLE model and CMIP6 climate projections. Environ Earth Sci. 2025;84(6). doi: 10.1007/s12665-025-12184-6

[64] Li Y, Xie W, Sui K, Zhang D, Wan Q. Revealing various change characteristics and drivers of ecological vulnerability in the Luan river basin based on the SRP model. Sci Rep. 2025;15(1):33021. doi: 10.1038/s41598-025-18564-z 41006679 PMC12475475

[65] Pal S, Sarda R. Damming effects on the degree of hydrological alteration and stability of wetland in lower Atreyee River basin. Ecological Indicators. 2020;116:106542. doi: 10.1016/j.ecolind.2020.106542

[66] Wang Z, Xiong H, Zhang F, Qiu Y, Ma C. Sustainable development assessment of ecological vulnerability in arid areas under the influence of multiple indicators. Journal of Cleaner Production. 2024;436:140629. doi: 10.1016/j.jclepro.2024.140629

[67] Cao C, Wang Y, Ding J, Li D. Study on the multi-scenario water resources vulnerability assessment and its influencing factors in the Kaidu River Basin considering ecological water demand. Journal of Hydrology: Regional Studies. 2025;62:102779. doi: 10.1016/j.ejrh.2025.102779

[68] Zhang X, Zheng Y, Yang Y, Ren H, Liu J. Spatiotemporal evolution of ecological vulnerability on the Loess Plateau. Ecological Indicators. 2025;170:113060. doi: 10.1016/j.ecolind.2024.113060

[69] Han H, Li H, Zhang K. Urban water ecosystem health evaluation based on the improved fuzzy matter-element extension assessment model: case study from Zhengzhou city, China. Mathematical Problems in Engineering. 2019;2019:7502342. doi: 10.1155/2019/7502342

[70] Wang X, Guo Y. Analyzing the consistency of comparison matrix based on G1 method. Chinese Journal of Management Science. 2012;:65–70.

[71] Diakoulaki D, Mavrotas G, Papayannakis L. Determining objective weights in multiple criteria problems: The critic method. Computers & Operations Research. 1995;22(7):763–70. doi: 10.1016/0305-0548(94)00059-h

[72] Das U, Behera B. Geospatial assessment of ecological vulnerability of fragile Eastern Duars Forest integrating GIS-based AHP, CRITIC and AHP-TOPSIS models. Geomatics, Natural Hazards and Risk. 2024;15(1). doi: 10.1080/19475705.2024.2330529

[73] Yuan X, Guo B. Dynamic Monitoring of the Ecological Vulnerability for Multi-Type Ecological Functional Areas during 2000–2018. Sustainability. 2022;14(23):15987. doi: 10.3390/su142315987

[74] Tang M, Li R, Dai X, Yu X, Cheng X, Yang S. New Electric Power System Stability Evaluation Based on Game Theory Combination Weighting and Improved Cloud Model. Sustainability. 2024;16(14):6189. doi: 10.3390/su16146189

[75] Dai R, Xiao C, Liang X, Yang W, Chen J, Zhang L, et al. Spatial-temporal evolution law analysis of resource and environment carrying capacity based on game theory combination weighting and GMD-GRA-TOPSIS model. Evidence from 18 cities in Henan Province, China. Journal of Cleaner Production. 2024;439:140820. doi: 10.1016/j.jclepro.2024.140820

[76] Gu W, Fu H, Jin W. Landscape Pattern Changes and Ecological Vulnerability Assessment in Mountainous Regions: A Multi-Scale Analysis of Heishui County, Southwest China. Land. 2025;14(2):314. doi: 10.3390/land14020314

[77] Lei J, Li C, Yang W, Yang X, Xing X, Feng X. Assessment of ecological drought vulnerability of the Qinghai-Tibet Plateau from 2003 to 2022 based on multi-source data. CATENA. 2024;247:108512. doi: 10.1016/j.catena.2024.108512

[78] Cao J-S, Yang Y-Q, Deng Z-Y, Hu Y-D. Spatial and temporal evolution of ecological vulnerability based on vulnerability scoring diagram model in Shennongjia, China. Sci Rep. 2022;12(1):5168. doi: 10.1038/s41598-022-09205-w 35338223 PMC8956742

[79] He S, Nong L, Wang J, Zhong X, Ma J. Revealing various change characteristics and drivers of ecological vulnerability in the mountains of southwest China. Ecological Indicators. 2024;167:112680. doi: 10.1016/j.ecolind.2024.112680

[80] Tang L, Hu X, Luo Z, Wei B, Wang Y, Zhang Y. Spatiotemporal coupling of ecological vulnerability and urbanization level and their interactive influencing factors: a case study of Hunan Province. Acta Ecologica Sinica. 2024;44:4662–77. doi: 10.20103/j.stxb.202307181532

[81] Luo K, Guan C, Liu Y, Li R, Wei G. Spatial-temporal evolution and scenario simulation of ecological vulnerability in the Yangtze River economic belt. Resources and Environment in the Yangtze Basin. 2026;35:417–27. doi: 10.11870/cjlyzyyhj202602011

[82] Wei Y, An M, Huang J, Fang X, Song M, Wang B, et al. How human activities affect and reduce ecological sensitivity under climate change: Case study of the Yangtze River Economic Belt, China. Journal of Cleaner Production. 2024;472:143438. doi: 10.1016/j.jclepro.2024.143438

[83] Lv Y, Li Y, Zhang Z, Luo S, Feng X, Chen X. Spatio-temporal evolution pattern and obstacle factors of water-energy-food nexus coupling coordination in the Yangtze river economic belt. Journal of Cleaner Production. 2024;444:141229. doi: 10.1016/j.jclepro.2024.141229

[84] Bi Y, Zheng L, Wang Y, Li J, Yang H, Zhang B. Coupling relationship between urbanization and water-related ecosystem services in China’s Yangtze River economic Belt and its socio-ecological driving forces: A county-level perspective. Ecological Indicators. 2023;146:109871. doi: 10.1016/j.ecolind.2023.109871

[85] Tang L, Zhou Z, Zhao H, Qian Z, Hou S, Liu B. Spatio-temporal evolution and influencing factors of the coupling coordination between water ecological resilience and new urbanization: a case study of the Yangtze River Economic Belt. Journal for Nature Conservation. 2026;89:127123. doi: 10.1016/j.jnc.2025.127123

[86] He L, Wang L, Lin A, Zhang M, Xia X, Tao M, et al. What drives changes in aerosol properties over the Yangtze River Basin in past four decades? Atmospheric Environment. 2018;190:269–83. doi: 10.1016/j.atmosenv.2018.07.034

[87] Ma Y, Wang W, Jin S, Li H, Liu B, Gong W, et al. Spatiotemporal variation of LAI in different vegetation types and its response to climate change in China from 2001 to 2020. Ecological Indicators. 2023;156:111101. doi: 10.1016/j.ecolind.2023.111101

[88] Bryan BA, Gao L, Ye Y, Sun X, Connor JD, Crossman ND, et al. China’s response to a national land-system sustainability emergency. Nature. 2018;559(7713):193–204. doi: 10.1038/s41586-018-0280-2 29995865

[89] Turner BL 2nd, Kasperson RE, Matson PA, McCarthy JJ, Corell RW, Christensen L, et al. A framework for vulnerability analysis in sustainability science. Proc Natl Acad Sci U S A. 2003;100(14):8074–9. doi: 10.1073/pnas.1231335100 12792023 PMC166184

